# The Effect of pH during Fabrication of Platinum-Containing Polymeric Arsenical Hydrogels

**DOI:** 10.1021/acs.macromol.5c01254

**Published:** 2025-07-17

**Authors:** Alexandros Magiakos, Spyridon Efstathiou, Evelina Liarou, Andrea Dsouza, Chrystala Constantinidou, Paul Wilson

**Affiliations:** † Department of Chemistry, 2707 University of Warwick, Coventry CV4 7AL, U.K.; ‡ Warwick Medical School, 2707 University of Warwick, Coventry CV4 7AL, U.K.

## Abstract

Intrinsic and acquired resistance, along with the systemic toxicity of platinum and arsenic therapeutics, necessitate the development of alternative chemistries and delivery strategies for Pt- and As-containing drugs. Stimuli-responsive hydrogels offer dynamic physicochemical adaptability, making them highly suitable for biomedical applications. Herein, we investigate the pH-responsive mechanical and antimicrobial properties of platinum-containing arsenical hydrogels. Poly­(*N,N*-dimethylacrylamide-*co*-4-(*N*-acrylamido)­phenylarsonic acid), P­(DMAm_0.92_-*co*-AsAm_0.08_), **P**_**As**_ was cross-linked with Pt^II^ (from K_2_PtCl_4_) under varying pH conditions to form hydrogels. Spectroscopic techniques (UV–vis, FT-IR, ^1^H, and ^195^Pt NMR) revealed that arsenic acid protonation influences Pt^II^–O–As^V^ interactions, impacting hydrogel integrity and dynamic behavior. Rheological analysis confirmed the pH-dependent mechanical properties, where increased pH strengthened metal–ligand interactions, enhancing material’s stiffness. Self-healing properties were demonstrated via strain recovery upon cutting for all materials, while resilience upon stretching was enhanced for the looser network environment under acidic conditions. Swelling studies indicated better stability in neutral environments, whereas increased ionic strength contributed to additional structural integrity. SEM-EDX confirmed morphological changes as a function of pH, corroborating the presence of both As and Pt under all pH conditions. The antimicrobial potential of these hydrogels was evaluated against Gram-positive (, ) and Gram-negative (uropathogenic , *K12 MG1655*) bacteria, demonstrating a similar—if not improved—antimicrobial profile in all cases compared to the individual components. This study advances the understanding of pH-modulated mechanical properties and antimicrobial activity of arsenic–platinum hydrogels, which are promising candidates for infection treatment or targeted drug delivery applications.

## Graphical abstract

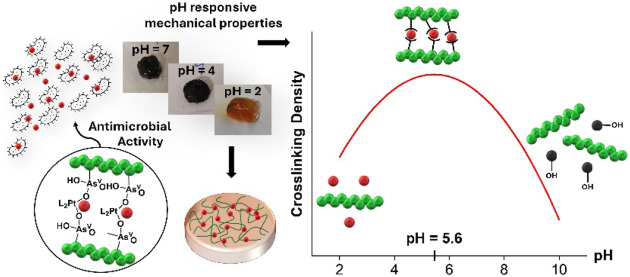

## Introduction

Hydrogels are water-absorbing polymeric networks resembling the inherent properties of natural living tissues.^1^ Based on their water content, they offer a wide range of physical, mechanical, and biochemical properties, making them invaluable materials in environmental,^2^ chemical,^3^ nanotechnology,^4^ and biomedical applications,^5^ including bioelectronics,^6^ biosensing,^7^ tissue engineering, contact lenses, drug delivery, antimicrobial treatments, and wound dressing.^8–11^ The nature and type of cross-linking significantly influence the physical and mechanical behavior of these materials and define their high versatility and tunability upon careful design.^12^ It is these characteristics that have garnered scientific interest in the development of stimuli-responsive hydrogels that can undergo reversible physicochemical changes in response to external stimuli, making them particularly useful for applications requiring dynamic control over material properties, drug release, or bioactivity.^13,14^

Stimuli-responsive hydrogels are often described as “smart materials,”^15^ highlighting their excellent ability to adapt their structure, shape, size, and properties in response to factors such as pH, temperature, ionic strength, redox conditions, magnetic and electric fields, light, enzymes, and others.^16,17^ The choice of polymer matrices and functional additives is essential for their sensitivity, response kinetics, and stability, as the chemical and physical bonds within the network can be reorganized or broken down through chemical, physical, hydrolytic, and enzymatic alterations.^18,19^ To this point, the design of pH-responsive hydrogels for biomedical applications is crucial, as many biological processes involving wound healing, tumor microenvironments, and bacterial infections are associated with localized pH variations.^20,21^ For instance, inflamed or infected tissues, depending on the stage of the infection, often exhibit acidic conditions,^22,23^ whereas extracellular environments in tumors can have lower pH levels than normal tissues.^24^ Thus, pH-modulated mechanical properties offer a route for usage in systems where upper or lower pH limits of stability are beneficial, such as controlled drug delivery.^25–27^

Various chemical strategies have been employed to engineer such materials, utilizing covalent, noncovalent, or hybrid cross-linking approaches.^28–30^ Additionally, polyelectrolytes have emerged as easily tunable materials whose sensitivity depends on their p*K*_a_ values.^31^ The ionization of pendant acidic or basic groups results in selective drug release, stability, and swelling. Numerous examples include poly­(carboxylic acid)­s, poly­(phosphoric acid)­s , and poly­(sulfonic acid)­s , as well as polyamines as basic groups.^32^

Metal–ligand coordination offers a particularly intriguing, noncovalent strategy for hydrogel fabrication, with a broad range of binding strengths possible. These can be fine-tuned by choosing appropriate ligands, metal centers, coordination geometries, and oxidation states. Unlike traditional networks, the dynamic metal–ligand coordination enables reorganization through solvent or ligand exchange, which offers excellent mechanical strength and self-healing properties. Their adaptability to different physiological conditions makes them attractive candidates for advanced biomedical systems, surpassing conventional pH-responsive hydrogel designs.^33–36^

Recently, we reported the development of a hydrogel platform based on polymeric arsenical interaction with Pt^II^ ions, primarily via metal coordination between oxygen atoms of the pendant arsenic acid group (As^V^), and secondarily through physical interactions.^37^ The nature of the soft acid–base binding of platinum to the arsenic polyelectrolyte enables the formation of soft yet strong hydrogels with tunable stiffness based on [Pt]/[As] mole ratios, high swelling capacity, and remarkable self-healing properties. The antimicrobial potential of these materials further underscores their biomedical significance.

Our interest in platinum (Pt) and arsenic (As) compounds is based on their extensive use in medicine.^38,39^ With cisplatin being first approved by the FDA for cancer treatment in 1976,^40^ carboplatin^41^ and oxaliplatin,^42^ alongside arsenic trioxide (As^III^)^43^ and darinaparsin^44^ are some examples widely recognized for their role in chemotherapy. Beyond oncology, Pt and As compounds exhibit a broad antimicrobial spectrum. Various organoarsenicals have been reported to treat bacterial infections, while cisplatin is known to inhibit bacterial growth.^45,46^ Nevertheless, systemic toxicity, as well as intrinsic and acquired resistance, are major problems associated with their action.^47,48^ Hence, alternative strategies involving the synergy of Pt and As in materials design have attracted scientific interest. For example, O’Halloran et al. introduced arsenoplatins as a new class of anticancer agents with greater bioactivity against solid tumors than their parent Pt^II^ and As^III^ drugs.^49–51^

The delivery of platinum drugs employing polymeric hydrogels is a known field that can significantly improve drug distribution, target selectivity, and stimuli-responsive release, reducing side effects and overall enhancing therapeutic efficiency.^52,53^ Chitosan-based hydrogels exploit the physical entrapment of platinum drugs,^54,55^ where carboxylic or sulfate-based hydrogels coordinate with oxaliplatin for pH-responsive release.^56^ Polymers containing amino acid moieties, such as histidine and phenylalanine moieties, are excellent examples of cisplatin delivery systems.^57^ Finally, metalloid-based polymers have shown affinity for Pt^II^ ions via metal-oxygen dynamic coordination bonds that stabilize the network and exhibit redox-responsive properties.^58^

Our group has previously demonstrated the diverse chemical reactions of polymeric arsenical scaffolds, which have been exploited for protein/peptide–polymer bioconjugation,^59^ fabrication of responsive polymeric arsenical nanoparticles^60–64^ and hydrogels^65^ that are nontoxic and able to support cell culture, as well as atomic-scale imaging of polymer properties.^66^

In this study, we build on our recent introduction of platinum-containing polymeric arsenical hydrogels to investigate the pH-responsive mechanical properties of these materials, with potential applications in the controlled delivery of both arsenic and platinum. The chemical interactions within this metal-polyelectrolyte system as a function of pH are explored, while the self-healing, mechanical, and swelling/stability properties across different pH levels are evaluated. Additionally, we assess the antimicrobial properties of the resulting hydrogels against Gram-positive and Gram-negative bacterial cell lines. Overall, this research serves as a step forward in understanding these multifunctional materials, with the aim of creating responsive therapeutic platforms for the targeted delivery of As and Pt in the battle against infectious and noncommunicable diseases.

## Materials and Methods

### Materials

Acryloyl chloride (≥97%), K_2_PtCl_4_, *N,N*-dimethylacrylamide (DMAm, containing 500 ppm monomethyl ether hydroquinone as an inhibitor), Na_2_HPO_4_, KH_2_PO_4_, NaH_2_PO_4_ and all deuterated solvents (D_2_O, CDCl_3_) were obtained from Sigma-Aldrich. NaClO_4_, Na_2_CO_3_, NaCl, NaOH, and KOH pellets; PBS 1X pH 7.4 (Gibco); concentrated HCl (37%); and H_2_SO_4_ (98%) were purchased from Fisher Scientific. Chloroform and HPLC-grade water were received from VWR International LLC. 2,2’-Azobis­[2-(2-imidazolin-2-yl)­propane] dihydrochloride (VA-044) was purchased from Wako Chemicals. Alfa Aesar provided *p*-arsanilic acid (≥98%). An ultrapure Milli -Q Type I water system was purchased from Merck. A Lucky Reptile Herp Nursery II Incubator equipped with an H–B Instrument Durac electronic thermometer-hygrometer was bought from Fisher Scientific. All reagents were used without further purification unless otherwise stated. Before any experiment, the hydrogels were washed three times with Milli-Q water to remove excess unreacted material stuck on the surface and dried thoroughly with paper tissue.

Uropathogenic*CFT073* (*UPEC*) and *K12 MG1655* were obtained from Dr Chrystala Constantinidou (Warwick Medical School, University of Warwick). *USA 300 JE2* and were obtained from Prof. Meera Unnikrishnan (Warwick Medical School, University of Warwick). Lysogeny Broth (LB), Tryptic Soy Broth (TSB), and Mueller Hinton Broth II (MHB-II) were purchased from Merck Millipore. Bacteriological agar was purchased from Becton Dickinson & Company Difco. Resazurin sodium salt was obtained from Acros Organics.

### General Procedure for the Synthesis of 4-(*N*-Acrylamido)­phenylarsonic Acid Monomer (AsAm)

Potassium hydroxide (5.1 g, 91.8 mmol) was dissolved in deionized water (125 mL). A mixture of *p-*arsanilic acid (10.0 g, 46.1 mmol) and Na_2_CO_3_ (14.5 g, 137.1 mmol) was added portionwise to the KOH solution to achieve dissolution. Acryloyl chloride (5.6 mL, 68.9 mmol) in dichloromethane (25 mL) was quickly added to the *p*-arsanilic acid solution at 0 °C and stirred for 15 min. The aqueous phase was collected and carefully acidified to pH 1 through the addition of H_2_SO_4_ (98%) which resulted in the precipitation of the product. The precipitate was collected by filtration, washed with cold water, and dried in a vacuum oven overnight to afford the title compound as a white solid (12.08 g, 49.9 mmol, α = 96.9%). ^**1**^ **H NMR** (D_2_O/NaOH 0.1 M, 400 MHz): δ_H_ (ppm) = 7.55 (2H, d, J_HH_ = 8.07 Hz, AsCC**H**), 7.11 (2H, d, J_HH_ = 7.95 Hz, CC**H**), 6.25 (1H, m, J_HH_ = 10.51, 6.85 Hz, CC**H**), 5.95 (1H, d, J_HH_ = 17.36 Hz, CH**H**), 5.53 (1H, d, J_HH_ = 10.51 Hz, C**H**H); ^**13**^ **C NMR**: δ_C_ (ppm) = 123.12 (**Ar**), 124.29 (H_2_ **C**=C−), 130.53 (**Ar**) 130.62 (H_2_C=**C**−), 133.34 (**Ar**), 134.73 (**Ar**), 168.72 (−**C**=O); **FT-IR** (ATR, cm^–1^): 3440, 3296, 3189, 3110, 3060, 2780, 1673 (*v*C=O), 1640, 1622, 1589, 1532, 1296, 906 (*v*AsO_*x*_), 834 (*v*AsO_*x*_), 777.

### General Procedure for Polymer Synthesis via Free Radical Polymerization (P_As_)

AsAm was solubilized in water with 1 equivalent of sodium hydroxide and added to a cooled aqueous solution of DMAm (1 M) containing VA-044 (0.01 equivalent with respect to total monomer content). The solution was degassed for 15 min before heating at 90 °C for 2 h. The full consumption of the monomer was confirmed by NMR before dialysis (SpectrumLabs PreWet Laboratory dialysis membrane, 6000 Da) and freeze-drying directly to yield white solids (<99%). ^**1**^ **H NMR** (D_2_O, 400 MHz): δ_H_ (ppm) = 7.20–7.82 (aromatic **H**), 2.3–3.2 (−NC**H**_3_, −C**H–** backbone), 1.0–1.9 (−CC**H**_**2**_C– backbone); **FT-IR** (ATR, cm^–1^): 3405, 3236, 2930, 1672 (*v*C=O Amide I—AsAm), 1605 (*v*C=O Amide I—DMAm), 1532 (*b*N–H Amide II—AsAm), 1254 (*v*C–N Amide III—DMAm), 880/862 (*v*AsO_*x*_), 719 (*b*, *v*AsOH/*p*-aromatic bending *Oop*).

### General Procedure for P­(DMAm_0.92_-*co*-AsAm_0.08_)-Pt (P_As_-Pt) Hydrogel Synthesis at Different pH Conditions

The pH of Milli-Q water was adjusted to the desired value using 0.1 M HCl or NaOH and measured with a pH meter. NaClO_4_ was added to the preadjusted aqueous solutions at a final constant concentration of 0.1 M in all cases to maintain a consistent ionic strength. Then, the P­(DMAm_0.92_-*co*-AsAm_0.08_) (**P**_**As**_) polymer scaffold was dissolved in the preadjusted pH solutions at a 10 wt % concentration (2.5 wt % for UV–vis measurement). The resulting solutions were left to incubate overnight in the dark at ambient temperature before the pH values were remeasured and readjusted with a few drops of HCl or NaOH (0.1 or 1 M). After incubation for another 24 h, the pH of the final solutions was remeasured (and readjusted where necessary) before adding the K_2_PtCl_4_ salt (1 eq. to AsAm) and incubating overnight at 50 °C. This resulted in the successful formation of black-orange colored hydrogels. Hydrogels at pH = 2, 4, 7, and 10 will be referred to in short as **P**_**4**_ **-Pt@2**, **P**_**4**_ **-Pt@4**, and **P**_**4**_ **-Pt@7** and **P**_**4**_ **-Pt@10**, respectively.

### Preparation of Phosphate Buffer Solutions

pH 2, 0.01 M, *I* = 0.12: 0.98 g of phosphoric acid was dissolved in approximately, 900 mL of HPLC-grade water, and 6.743 g of NaCl was added to the solution. A few drops of 37% HCl were used to adjust the pH before making up the volume to 1 L pH 4, 0.05 M, *I* = 0.12: 5.04 g Na_2_HPO_4_ and 3.01 g KH_2_PO_4_ were dissolved in approximately 900 mL of HPLC-grade water. A few drops of 0.2 M HCl or glacial acetic acid were utilized to adjust the pH before making up the volume to 1 L pH 7, 0.05 M, *I* = 0.12: 1.1 g NaH_2_PO_4_ were dissolved in approximately 900 mL of HPLC-grade water, and 5.749 g of NaCl was added to the solution. A few drops of 0.1 M HCl or NaOH were utilized to adjust the pH before making up the volume to 1 L.

*Safety* All experiments involving *p*-arsanilic acid and its derivatives were conducted in a fume hood with appropriate PPE, and waste was disposed of according to institutional hazardous waste protocols to ensure safety and compliance with regulations.

### Nuclear Magnetic Resonance (^1^H NMR, ^13^C NMR, ^195^Pt NMR)

All spectra were recorded on Bruker DPX-400 and DPX-600 MHz spectrometers in deuterated chloroform (CDCl_3_) or deuterium oxide (D_2_O). A Bruker triple resonance observe TXO CryoProbe was used to acquire ^195^Pt NMR. Chemical shifts are reported in ppm relative to the internal standard tetramethylsilane (TMS). ^195^Pt NMR chemical shifts were referenced indirectly to TMS in the ^1^H NMR spectrum such that K_2_^195^PtCl_6_ in D_2_O would resonate at 0.0 ppm (δ = −1617 ppm (s, ^195^Pt) for K_2_PtCl_4_). ACD/Laboratories software was used to analyze all data. For NMR measurements, hydrogels were prepared inside the NMR tube as follows: **P**_**As**_ **-Pt** solutions (10 wt %) at different pH conditions were prepared inside a vial following the described process and subsequently transferred into the NMR tubes, where they were left at 25 °C for 5 days to ensure the formation of stable hydrogels and minimize the presence of platinum hydroxides (Figure S3).

### Infrared Spectroscopy (IR)

All infrared spectra were recorded using a Bruker ALPHA II or Agilent Cary 630 Fourier-transform infrared spectrometer (FT-IR), scanning between wavenumbers of 400 and 4000 cm^–1^.

### Size Exclusion Chromatography (SEC)

Aqueous SEC: The Agilent PL50 instrument was equipped with a differential refractive index (DRI) detector. The system was equipped with a PL Aquagel OH 60 column (300 mm × 7.5 mm) and an 8 μm Aquagel guard column. The eluent was 80% NaNO_3_ in Milli-Q water at 0.1 M and 20% methanol, with a flow rate of 1 mL·min^–1^ at 35 °C. PEG/PEO standards in the range of 190–1,100,000 g·mol^–1^ (Agilent *EasiVials*) were used for calibration. Prior to sample injection, the samples were filtered through a GVHP membrane with a 0.45 μm pore size. Experimental molar mass (*M*_n_, SEC) and dispersity (*Đ*m) values of the synthesized polymer were determined by conventional calibration using Agilent SEC software.

### UV–Visible Spectroscopy

UV–vis spectra were recorded on an Agilent Technologies Cary 60 UV–vis spectrometer in the range of 200–600 nm, equipped with a Quantum Northwest temperature-controlled cuvette holder to adjust the temperature during kinetic studies. For all measurements, BRAND semimicro polystyrene cuvettes with a 1 cm optical length, obtained from Sigma-Aldrich, were used. All gels were prepared inside the cuvette.

### Rheology

Rheological measurements were performed using an Anton Paar MCR 302 rheometer equipped with a parallel plate configuration (25 mm diameter, 1 mL scale). The data were analyzed using RheoCompass software and plotted with OriginLab software. The normal force was maintained at a constant 5 N during measurements, and all measurements were conducted at room temperature. For the frequency sweep, a constant strain (γ = 10.0%) was applied, and the frequency was ramped logarithmically from ω = 1 (or 0.1) to ω = 100 rad·s^–1^. Amplitude sweeps were performed at an oscillating frequency of ω = 10 rad·s^–1^. Cyclic strain experiments were conducted to examine self-healing behavior. For these tests, hydrogels were cut in half and recombined for 1 h in a humidity chamber before amplitude sweeps were remeasured. Each test was repeated at least 3 times for each sample.

### Scanning Electron Microscopy (SEM) and Energy-Dispersive X-Ray (EDX) Spectroscopy

Scanning electron microscopy was performed using a ZEISS Gemini SEM Field Emission Scanning Electron Microscope and a ZEISS Supra SEM. The best results were obtained when using the InLens detector with a ∼ 3.5 mm working distance, a 20 μm aperture (Gemini) or a 30 μm aperture (Supra), and an acceleration voltage of 1–7 kV, depending on sample tolerance. EDX spectroscopy and elemental analysis were performed using the Gemini instrument equipped with an SDD EDX detector. Gels, washed with H_2_O and then freeze-dried, were cast on carbon tabs (9 mm) attached to aluminum specimen stubs. To improve sample imaging, gold (Au) sputter coating was applied for 10 s prior to imaging. Elemental analysis was carried out during SEM imaging on the same samples using an Oxford Instruments X-Max 150 large-area SDD electron-dispersive X-ray analysis (EDX) detector.

### Swelling Studies

Freshly prepared hydrogels (10 wt %) were immersed in Milli-Q water or in different pH phosphate buffer/ionic strength solutions and left in a humidity chamber for 7 days at 25 °C. All hydrogels were periodically removed from the solution, excess water was removed using tissue, and the weight (W_s_) was recorded. The swelling ratio was calculated using the formula:

1 $$\mathrm{Swelling Ratio}\;(\%)=\frac{W_{s}-W_{o}}{W_{o}}\times 100$$

All experiments were performed at least twice, and results are presented with standard deviation bars. Solvent was added occasionally to maintain the volume at a constant 8 mL.

### *In Vitro* Antibacterial Assessment

The antibacterial activity of hydrogels was tested against Gram-negative (*UPEC* and *K12 MG1655)* and Gram-positive ( and ). Bacterial fluorescence intensity was measured using a BMG Labtech FLUOstar Omega microplate reader with a range of 240–740 nm.

*Antibiotic Diffusion Assay*. Bacteria were revived from glycerol stocks and streaked on LB agar (for and ) and Tryptic Soy Agar (TSA) (for ). Single bacterial colonies were picked from each agar plate and inoculated into LB (for and ) and TSB (for ) for an overnight primary culture. Subsequently, 100 μL of *UPEC*, *K12 MG1655*, and were plated onto LB agar plates, while was plated onto TSB agar plates, each as lawn cultures. The assay included three control samples: a **P**_**As**_ polymer scaffold aqueous solution at a concentration of 100 mg/mL (as control), a sodium perchlorate (NaClO_4_ 0.1 M) salt solution (salt control), and a platinum (Pt^II^) aqueous solution of 30 mM K_2_PtCl_4_, matching the Pt^II^ concentration in the 10 wt %, gel matrix (Pt control). The test samples comprised two hydrogels, **P**_**As**_ **-Pt@4** and **P**_**As**_ **-Pt@7**, to evaluate their effects on bacterial growth. They were cut into 0.4 cm diameter discs and placed on plates containing lawn bacterial growth. After hydrogel introduction on agar plates, the plates were incubated at 37 °C for 18 h, followed by measurement of the zone of bacterial inhibition. **P**_**As**_ **-Pt@2** gel was not used for this assay, as these hydrogels appeared to have poor physical stability based on visual observation, making it difficult to obtain defined 0.4 cm diameter hydrogels and thereby limiting quantitative measurement of the zone of bacterial inhibition.

*Bacterial Viability Assay.* Resazurin assay was used to determine bacterial viability upon exposure to arsenic–platinum hydrogels. The assay consisted of As, Pt, and salt controls, as described in the antibiotic diffusion assay. The test samples included **P**_**As**_ **-Pt@2**, **P**_**As**_ **-Pt@4**, and **P**_**As**_ **-Pt@7** hydrogels. Overnight primary bacterial cultures were prepared as described above. A stock solution of resazurin was prepared by dissolving 0.05 g of resazurin powder in 10 mL of PBS. All experiments were carried out in sterile 24-well plates. Two milliliters of cation-adjusted Mueller Hinton Broth II (MHB-II) was introduced into each well, followed by the addition of 10 μL of stock resazurin solution to yield a final concentration of 25 μg·mL^–1^. Wells containing MHB-II and resazurin were used as negative controls. Prior to the antibacterial assay, the three control samples and all hydrogels were sterilized by exposing them to ultraviolet radiation (BIO-LINK BLX-254, 80 W) for 10 min. The UV-sterilized gels were introduced into negative controls to observe any bacterial growth or contamination. The absence of bacterial growth or contamination indicated successful UV sterilization of the hydrogels. Wells containing MHB-II and resazurin with bacteria were used as positive controls. 0.7 g of each hydrogel was separately introduced into test wells consisting of MHB-II and resazurin. In positive controls and test wells, the overnight bacterial cultures were introduced such that the starting optical density (OD) of bacteria at 600 nm was 0.01. The plate was incubated at 37 °C and 110 rpm for 6 h, and fluorescence measurements at 585 nm were collected every 2 h over a period of 6 h using a BMG Labtech FLUOstar Omega plate reader.

### Reverse Phase High-Performance Liquid Chromatography (RP-HPLC)

RP-HPLC was performed on an Agilent 1260 Infinity Series stack equipped with a 1260 Quat Pump VL, a degasser, and a fraction collector (FC-AS). Samples were injected using an Agilent 1260 autosampler with a 5 mL injection (draw and injection rate of 800 μL·min^–1^). The RP-HPLC was fitted with a Phenomenex Luna C_18_ column (250 × 4.6 mm) with 5-μm packing (100 Å). Detection was achieved using an Agilent 1260 variable wavelength detector connected in series, with UV detection monitored at 320 nm. The total flow rate was set to 1.0 mL·min^–1^ and the temperature of the column was set to 20 °C. The mobile phase conditions were: A: 100% water with 0.04% v/v TFA, and B: 100% MeCN with 0.04% v/v TFA. The method followed is shown in Table 1.

**Table 1:** HPLC Standard Analysis Method

Time (min)	A (%)	B (%)
0	95	5
10	70	30
21	5	95

## Results and Discussion

### Polymer and Hydrogel Synthesis

The synthesis and characterization of the polymeric arsenical scaffold used in this work were reported in our recent publication introducing platinum-containing polymeric arsenical hydrogels.^37^ In this study, we elected to use P­(DMAm_0.92_-*co*-AsAm_0.8_) (**P**_**As**_) with an *M*_n_ = 42000 g·mol^–1^, *M*_w_ = 365,000 g·mol^–1^, and *Đ*_m_ = 8.84 due to its favorable long-term stability and mechanical properties under standard hydrogel fabrication conditions (Figure S1).

To investigate the impact of pH on hydrogel structure and mechanical properties, **P**_**As**_ (10 wt %) was dissolved in 0.1 M solutions of NaClO_4_ at pH 2, 4, 7, and 10. These solutions were mixed with K_2_PtCl_4_ before heating at 50 °C overnight to promote hydrogel formation (Figure 1). The pH values were selected based on the p*K*_a1_ (3.88) of the **P**_**As**_ pendent arsenic acid groups, which was determined by potentiometric titration in our previous work.^37^ Considering that ionic strength is an important factor that can influence metal–polymer interactions, it was kept constant (*I* = 0.1 M) across all pH values^67^ to enable a direct comparison of the results. NaClO_4_ was chosen to adjust the ionic strength, as the large ClO_4_^–^ ions do not compete for Pt^II^ coordination.

**Figure 1:**
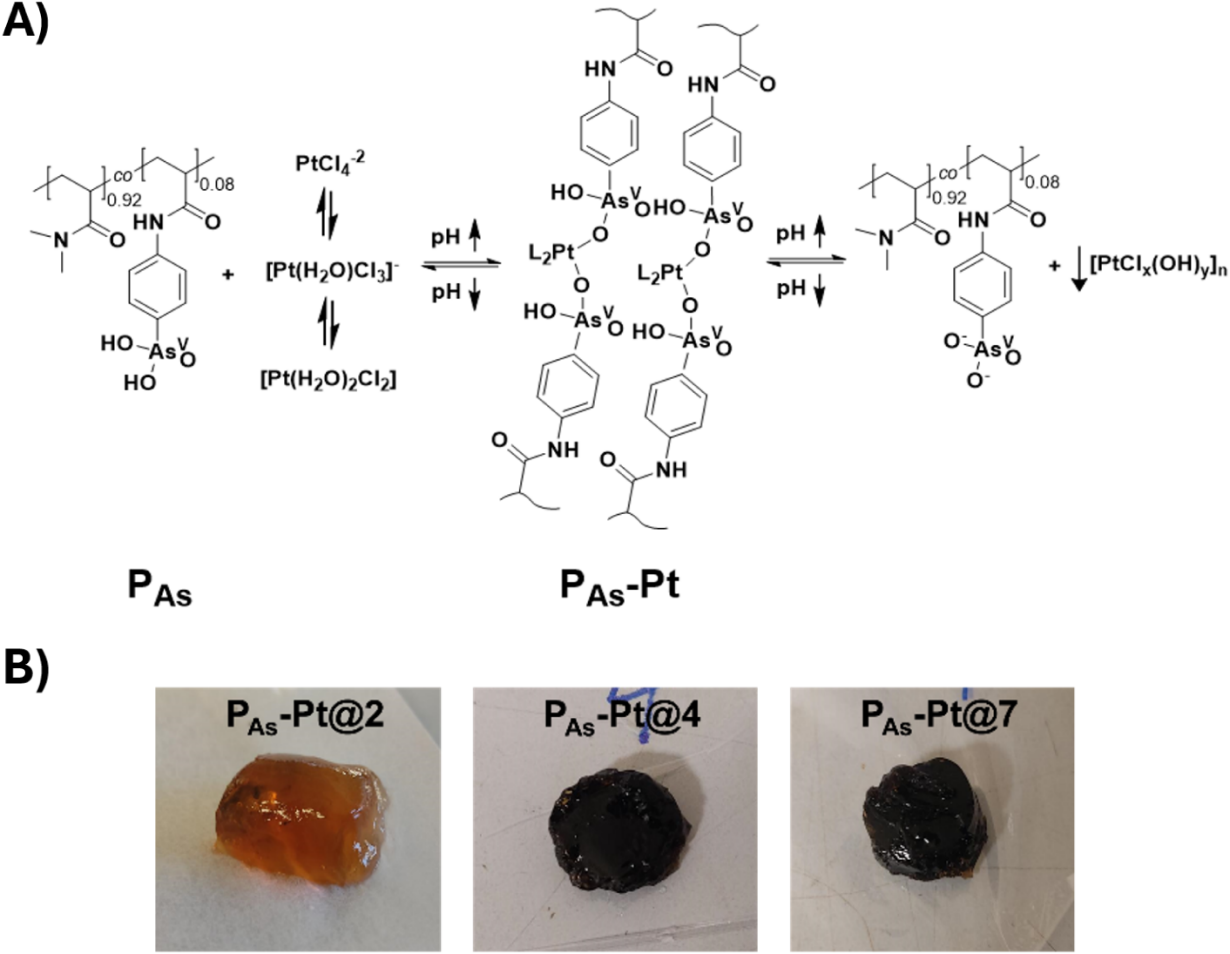
Structural features and pH-responsive behavior of platinum-containing polymeric arsenical (**P**_**As**_) hydrogels formed *via* direct complexation of Pt^II^ with arsenic acid pendant groups. (A) The coordination between Pt^II^ and arsenic acid is influenced by the acid/base equilibria of both the **P**_**As**_ polymer and the PtCl_4_^–2^ precursor. Deprotonation of the first hydroxyl group of the arsenic acid pendants (AsO_2_HOH → AsO_2_HO^–^) has a p*K*_a1_ = 3.88, while the hydrated Pt^II^ species either complexed or free undergo deprotonation at alkaline pH, with a reported p*K*_a_ = 7.33, forming dark precipitates in form of [PtCl_*x*_(OH)_*y*_]_n_ even in the presence of a stabilizing polymers such as the **P**_**As.**_ These equilibria were previously confirmed via potentiometric titrations.^37^ (B) Macroscopic images show hydrogel samples prepared at constant ionic strength (*I* = 0.1 M) and pH = 2, 4, and 7 (**P**_**As**_ **-Pt@2**, **P**_**As**_ **-Pt@4**, and **P**_**As**_ **-Pt@7**, respectively).

### Metal–Polymer Interaction at Different pH Environments

The hydrogel formation of **P**_**As**_ solutions (2.5 wt %) under different pH values in the presence of K_2_PtCl_4_ ([Pt]/[As] = 1) was evaluated by measuring UV–vis absorption after 24 h of incubation at 50 °C. A lower concentration of 2.5 wt % was selected in this experiment to reduce turbidity-related signal loss after gelation and ensure interpretable data within the range of the instrument. As reported in our previous work, hydrogel formation was indicated by a substantial increase in absorption across all wavelengths due to signal loss from turbidity effects.^68^ At pH 4 and pH 7, the solutions displayed a pronounced increase in absorption, confirming hydrogel formation (Figures 2B,C and S2). In contrast, the 2.5 wt % solution at pH 2 exhibited a minimal increase in absorption after 24 h (Figure 2A). This indicates that at lower concentrations (2.5 wt % rather than 10 wt %) and low pH (pH = 2), the **P**_**As**_ scaffold lacks sufficient interactions to support stable cross-linking (Figure S2). At pH 10, the solution failed to form a hydrogel, and a noticeably darker solution was obtained (Figures 2D and S2) that settled into a black precipitate. This phenomenon is attributed to the formation of platinum hydroxides. In aqueous solution, K_2_PtCl_4_ dissociates into [PtCl_4_][Bibr ref2] which is square planar and is easily hydrated to PtCl_3_(H_2_O)^−^ and PtCl_2_(H_2_O)_2_. In the absence of stabilizing agents (polymers in our case), these hydrated species can undergo deprotonation and subsequently oligo/polymerization to [PtCl_*x*_(OH)_*y*_]_n_ species, referred to as Pt­(OH)_2_-containing species.^69^ In our previous work, we presented the titration of 30 mM K_2_PtCl_4_, comparable in concentration to the gel system, revealing a clear inflection point at alkaline pH corresponding to a p*K*_a_ of 7.33. This inflection point remains unchanged in the presence of polymeric arsenicals (**P**_**As**_) at various [As]/[Pt] ratios, indicating that complexation does not affect the deprotonation of hydrated Pt^II^. Furthermore, the second deprotonation step of the arsenic acid moiety (p*K*_a2_ = 8.51) also remains unaffected, confirming that the second As–OH groups are not involved in As–O–Pt coordination.^37^ These observations indicate that at alkaline pH, hydroxide formation is a dominant process and disrupts As–O–Pt coordination, thereby destabilizing the hydrogel network.

**Figure 2:**
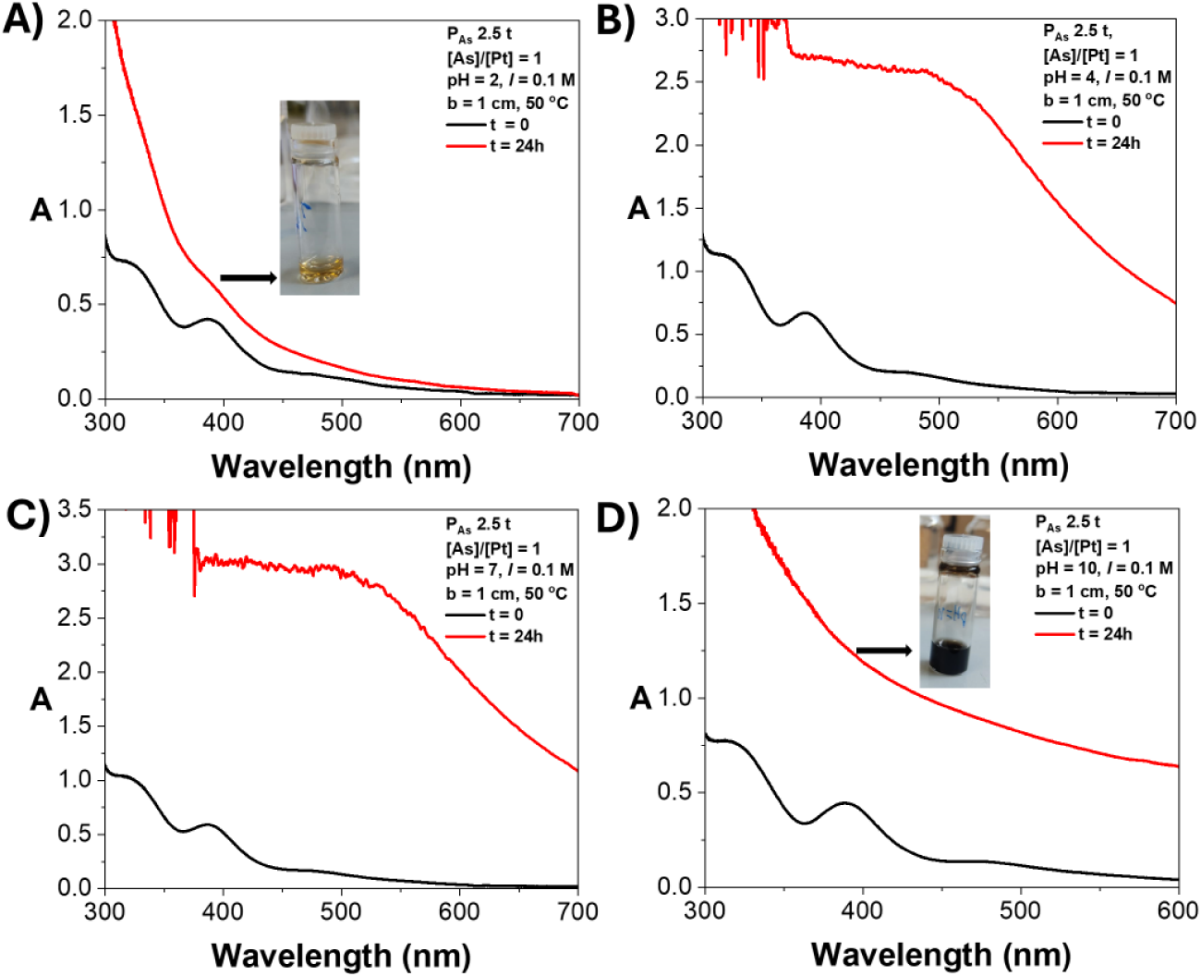
UV–vis absorption (300–700 nm) of **P**_**As**_/K_2_PtCl_4_ solutions (2.5 wt %) under different pH conditions after incubation at 50 °C for 24 h. (A) pH = 2, (B) pH = 4, (C) pH = 7, (D) pH = 10.

To investigate As–Pt interactions at various pH levels, 10 wt % **P**_**As**_ **-Pt** hydrogels were prepared for ^195^Pt and ^1^H NMR spectroscopy (see Materials and Methods section for sample preparation). In ^195^Pt NMR spectroscopy, all samples exhibited two clear peaks at −1617 ppm and −1184 ppm, which can be assigned to K_2_PtCl_4_ and [Pt­(H_2_O)­Cl_3_]^−^, respectively (Figure 3A).^70^ Interestingly, the intensity of the peaks decreased with increasing pH. Pt^II^ coordinates more efficiently to the pendant arsenic acid groups after deprotonation; hence, at low pH, a greater proportion of uncomplexed Pt^II^ species is present in acidic solution, resulting in a larger signal-to-noise ratio. At pH 10, platinum hydroxide formation resulted in a noisy spectrum lacking distinct peaks (Figures S3 and S4).

**Figure 3:**
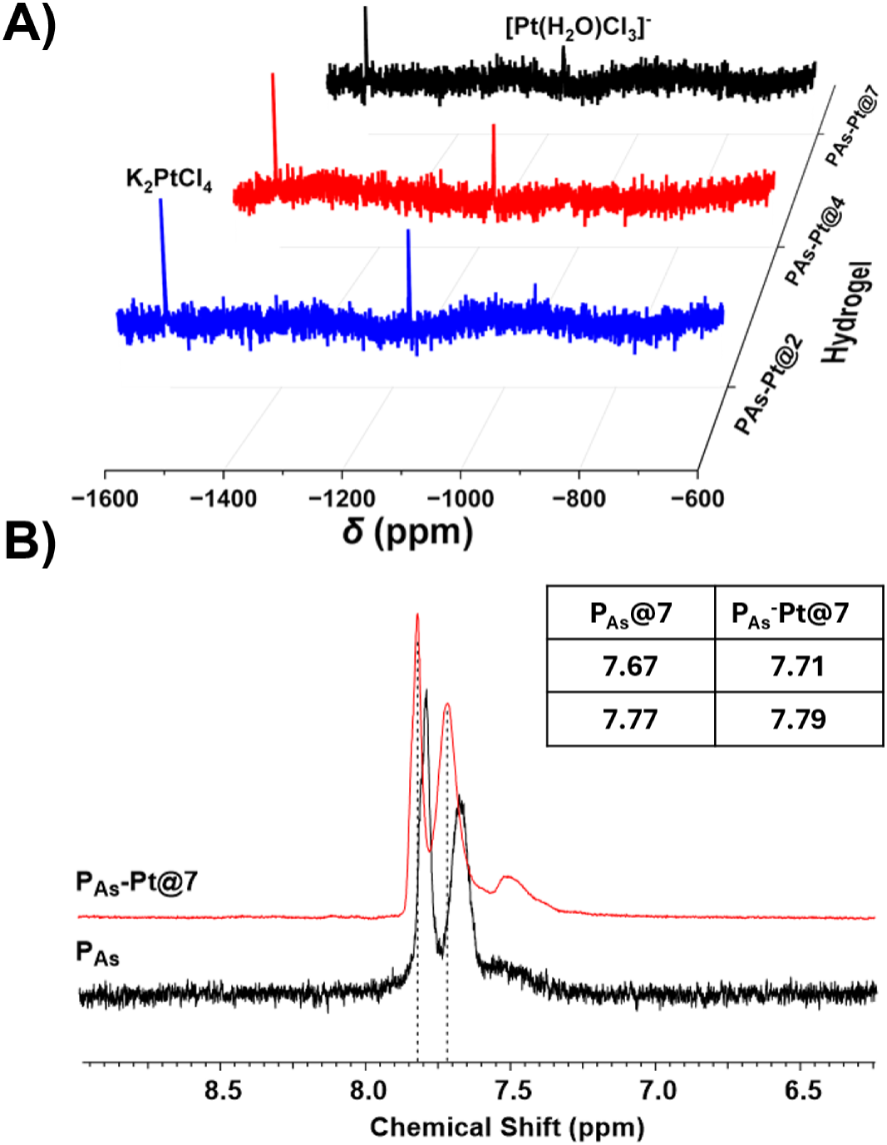
(A) ^195^Pt NMR (600 MHz, D_2_O) of **P_As_-Pt@2**, **P_As_-Pt@4**, and **P_As_-Pt@7** hydrogels in the range of −1600 to −600 ppm. (B) ^1^H NMR (400 MHz, D_2_O) of the corresponding hydrogels showing a downfield shift in AsAm side chain peaks upon Pt^II^ coordination.

^1^H NMR spectroscopy showed the characteristic peaks for the protons of the 1,4-substituted aromatic ring of the AsAm side chain of the free polymer scaffolds and upon As–O–Pt interaction when gelation was performed in an NMR tube. For the uncoordinated **P**_**As**_ it is known that the aromatic protons undergo a discrete downfield shift upon formation of the acid form (from the conjugate base) of arsenic acid.^37^ Comparison of the P_As_ at pH = 7 (**P**_**As**_ **@7**) and **P**_**As**_ **-Pt@7** indicated that As–O–Pt coordination resulted in a similar discrete downfield shift of the aromatic protons from 7.77/7.67 to 7.79/7.71 ppm (Figure 3B). At lower pH, the downfield shift of both **P**_**As**_ and **P**_**As**_ **-Pt** increases and does not change below the p*K*_a1_ of the arsenic acid group (Figure S5). This is consistent with other metal-coordinated supramolecular hydrogels, which are known to experience *gel-to-sol* phase transitions in acidic pH media due to changes in the ionization of the acidic groups.^71,72^

A detailed analysis of the FT-IR spectra of freeze-dried gels (Figure 4) revealed a shift in the As–OH stretching vibration from a single peak at 719 cm^–1^ (**P**_**As**_ **-Pt@4** and **P**_**As**_ **-Pt@7**) to a double peak at 728 cm^–1^ (v_s_) and 752 cm^–1^ (v_as_) after protonation (**P**_**As**_ **-Pt@2)**.^73^ Additionally, a broad peak emerging at 922 cm^–1^ for **P**_**As**_ **-Pt@2** is attributed to a single localized As=O bond energy, indicative of full protonation of arsenic acid groups. This As=O bond exhibits an asymmetrical stretching vibration at 833 cm^–1^, overlapping with the As–O–Pt vibration at 832 cm^– 1^, which exists in all hydrogels and is consistent with efficient cross-linking. Deprotonated As–O groups reside either in a complexed (As–O–Pt) or uncomplexed state, giving rise to distinct delocalization of As=O bond charges, resulting in additional vAs-O_*x*_ peaks at 888 cm^–1^ and 864 cm^–1^ for **P**_**As**_ **-Pt@4** and **P**_**As**_ **-Pt@7**, unlike **P**_**As**_ **-Pt@2** gel.^74,75^

**Figure 4:**
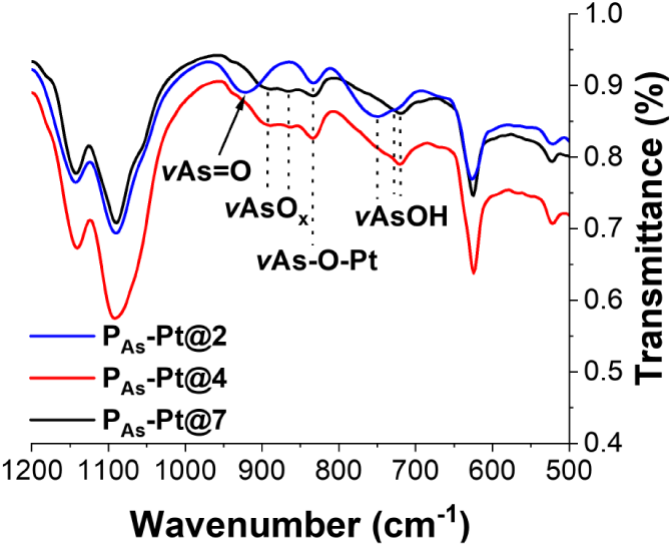
FT-IR spectra of **P**_**As**_ **-Pt@2**, **P**_**As**_ **-Pt@4**, and **P**_**As**_ **-Pt@7** hydrogels after lyophilization, showing changes to the AsAm functional group across different pH environments.

### pH-Responsive Mechanical Properties

The rheological properties of the hydrogels were examined by using dynamic oscillatory rheology. Frequency sweeps at a constant strain of γ = 10% (LVE region) revealed frequency-dependent loss modulus (*G″*), indicating a dynamically cross-linked system,^76^ which we propose is primarily stabilized by As–O–Pt coordination along with a contribution from complementary noncovalent interactions (Figure 5A).

**Figure 5:**
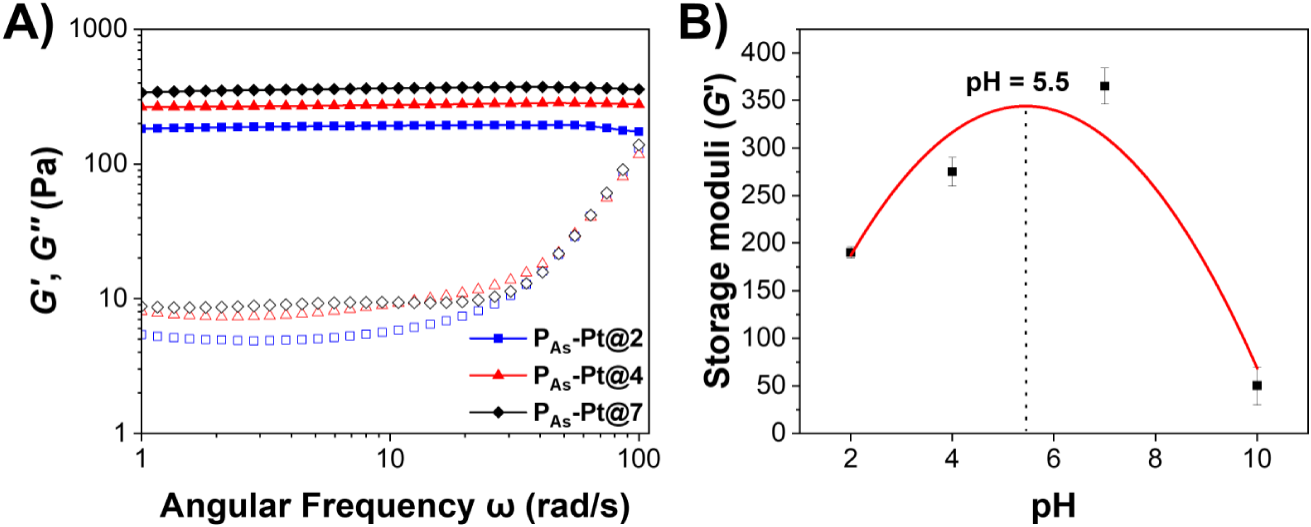
(A) Frequency sweep curves (ω = 1–100 rad·s^–1^) at 25 °C and at a constant strain of 10.0% for **P**_**As**_ **-Pt@2**, **P**_**As**_ **-Pt@4**, and **P**_**As**_ **-Pt@7** hydrogels obtained by plate-to-plate oscillatory rheology. On all occasions, storage modulus (*G’*, filled symbols) and loss modulus (*G″*, unfilled symbols); (B) *G’* at angular frequency of ω = 1 rad·s^–1^ as a function of pH showing the highest moduli at pH = 5.5. Red Gaussian is drawn for exemplification.

Interestingly, the storage modulus (*G’*) was found to be independent of the frequency. This observation provided an opportunity to further understand the effect of pH on the cross-linking junctions by measuring the cross-linking density of each hydrogel. Based on James and Guth’s rubber elasticity theory,^77^ the equilibrium shear modulus *G*_*e*_ can be obtained as a constant value from the plateau of *G’* in frequency sweep curves, when *G″* is much smaller than *G’*. *G*_*e*_ is related to the cross-linking density (*N*) of the network according eq 2:

2 $$N=\frac{G_{e}}{RT}$$

Storage modulus *G’* and cross-linking densities *N* for **P**_**As**_ **-Pt** hydrogels at different pH are presented in Table 2. The dependence of the cross-linking density on the pH value supports the responsive nature of the interaction between Pt^II^ and arsenic acid groups (Figures 5B and S6). In particular, *G’* is lowest for **P**_**As**_ **-Pt@2**, where the pH is below the p*K*_a1_ of the **P**_**As**_ scaffold, and the highest proportion of the arsenic acid groups are in their acid form. Conversely, when the pH is above the p*K*_a1_, *G’* increases, reaching a theoretical maximum at pH 5.5. Above pH 7, *G’* decreases again, with an unstable gel forming at pH 10 (**P**_**As**_ **-Pt@10**), exhibiting *G’* = 51 Pa at ω = 1 rad·s^–1^ (130 Pa at ω = 100 rad·s^–1^). This instability is attributed to the tendency to form platinum hydroxides within the network, as indicated by the material’s dark color (Figure S7). Moreover, *N* values verify the soft nature of the metal-coordinated network, unlike the higher values reported for harder covalently cross-linked networks.^78–80^

**Table 2:** Storage Modulus *G’* (Constant Modulus Region) and Calculated Crosslinking Density for P_As_-Pt Hydrogels Fabricated at Different pH Conditions

Hydrogel	*G’* (Pa)	Cross-linking density *N* (mol/m^3^)
**P**_**As**_ **-Pt@2**	190 ± 6	0.077
**P**_**As**_ **-Pt@4**	275 ± 15	0.11
**P**_**As**_ **-Pt@7**	365 ± 19	0.15

The kinetic flexibility of polymer chains within this physical network is influenced by the reorganization of Pt^II^ coordination under applied stress *via* a solvent-mediated ligand exchange mechanism.^81^ Surprisingly, the *G’* frequency independence is antithetical to physically cross-linked hydrogels,^82,83^ indicating a constraint of the reorganization phenomena in the case of the examined materials. A possible explanation for this is the presence of Na^+^ at concentrations that are more than 3 times greater (0.1 M) than Pt^II^. The Na^+^ can screen the negative charges of the arsenic acid groups present when pH > p*K*_a1_, hindering the potential for remodeling of the platinum coordination.^84^ As a result, the charge-shielding effect of counterions leads to a decreased flexibility of the polymer chains, providing additional structural integrity and stability.

To investigate the effect of Na^+^ concentration on gel stability, a series of **P**_**As**_ **-Pt** gels were fabricated in Milli-Q water by mixing 10 wt % solutions of **P**_**As**_ with K_2_PtClt_4_ ([As]/[Pt] = 1) and incubating at 50 °C overnight. The resulting gels were separately immersed in Milli-Q water containing different concentrations of NaClO_4_ and their swelling behavior was investigated (Figure S8A). All gels maintained integrity and reached swelling equilibrium after 120 h, with maximum swelling ratios decreasing as the Na^+^ concentration increased (Figure S8B). Na^+^ ions diffuse into the gel, screening negative charges on arsenic acid groups, limiting water ingress and exchange, and reducing chain mobility. Additionally, higher Na^+^ levels lower the osmotic pressure gradient, further restricting solvent uptake.^85,86^ Overall, the presence of Na^+^ enhances the gel’s structural stability *via* electrostatic interactions, in agreement with rheological analysis.

Considering the impact of pH on the mechanical properties of the materials, macroscopic self-healing tests and recycled oscillatory strain sweep experiments (at a constant frequency of ω = 10 rad·s^–1^) were conducted after cutting the hydrogels and recombining them for 1 h in a humidity chamber at room temperature (Figure S9). The amplitude sweeps did not reach a crossover point, indicating no collapse of the network upon large deformation. The samples successfully healed into free-standing hydrogels and regained their initial *G’* and *G’’* values. The physical nature of the system enables polymer chain migrations and the reformation of coordination and physical bonds, resulting in a complete reformation of the network after damage.^3^ Qualitative stretching of the healed gels was performed, and resilience was recorded (Figure S10 and Videos SV1, SV2). The lower cross-linked **P**_**As**_ **-Pt@2** gels exhibited remarkable resilience to stretching, while **P**_**As**_ **-Pt@4** and **P**_**As**_ **-Pt@7** showed limited resilience, eventually breaking at the original point of incision. Reflecting the mechanical properties of the materials, less rigid networks exhibit greater overall polymer chain mobility, allowing gels to undergo easier reformation of physical interactions.

A cross-section of freeze-dried hydrogels was imaged using scanning electron microscopy (SEM) to investigate the effect of pH during hydrogel formulation on the internal morphology of the hydrogels (Figure 6). The looser **P**_**As**_ **-Pt@2** network exhibits a more irregular and open structure, while the stiffer **P**_**As**_ **-Pt@4** and **P**_**As**_ **-Pt@7** display a more organized structure. The characteristic peaks attributed to As and Pt were detected through energy-dispersive X-ray (EDX) spectroscopy during SEM imaging, corroborating their presence on the surface of all gels (Figure S11A–C).

**Figure 6:**
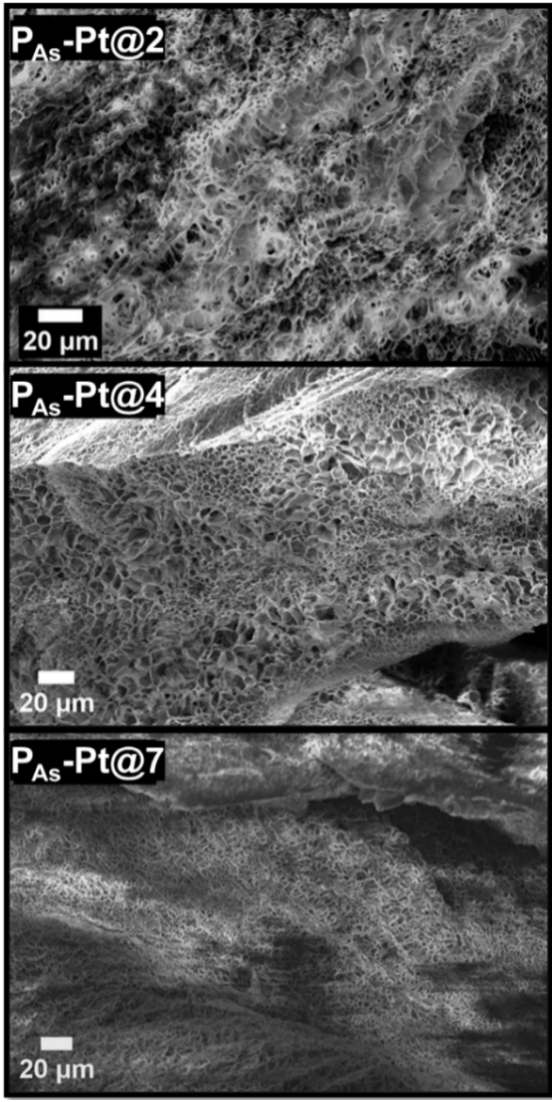
Scanning electron microscopy (SEM) of dry **P**_**As**_ **-Pt@2, P**_**As**_ **-Pt@4, and P**_**As**_ **-Pt@7** hydrogels after lyophilization (scale bar = 20 μm).

The polyelectrolyte nature of polymeric arsenical hydrogels makes their stability critically dependent on pH values, whereas swelling behavior defines their permeability and potential Pt^II^ delivery profile. Thus, the swelling properties were investigated separately by immersing freshly prepared gels in either Milli-Q water or PBS and measuring the swelling ratio over a period of 1 week (Figure 7 and Table S1). At low pH (**P**_**As**_ **-Pt@2**), the fully acidic form of the arsenic acid groups results in weaker As–O–Pt interactions. Consequently, the deep penetration of water leads to fragmentation after a few hours and complete collapse of the **P**_**As**_ **-Pt@2** hydrogel network after 120 h (Figure S12). Conversely, at pH values above the p*K*_a1_, (**P**_**As**_ **-Pt@4** and **P_As_-Pt@7**) the partially ionized arsenic acid groups form more stable As–O–Pt interactions and can retain their structural integrity, reaching equilibrium swelling after 120 h. **P**_**As**_ **-Pt@4** gel achieved the highest swelling degree of 1825% at 168 h, while **P**_**As**_ **-Pt@7** reached 1595%, reflecting the mechanical properties of the gels, where the higher cross-linked **P**_**As**_ **-Pt@7** displays less available space between polymer chains and limited chain flexibility, reducing its swelling capacity.^87,88^

**Figure 7:**
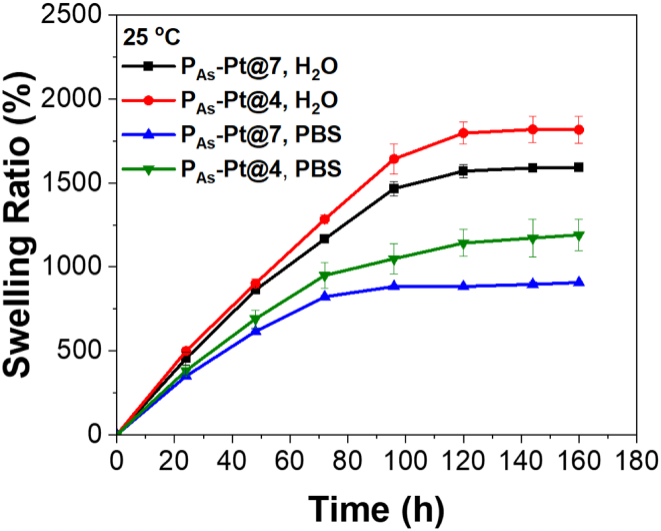
Swelling ratio curves of **P**_**As**_ **-Pt@4** and **P**_**As**_ **-Pt@7** hydrogels in Milli-Q water and PBS (0.01 M, *I* = 0.15 M, pH 7.4).

The swelling behavior in PBS showed the same trend, but there was a decrease in the equilibrium swelling ratios of both **P**_**As**_ **-Pt@4** and **P**_**As**_ **-Pt@7** compared to pure water. The maximum swelling degree was 1190% for **P**_**As**_ **-Pt@4** and 905% for **P**_**As**_ **-Pt@7**. The elevated salt concentrations in the PBS surrounding environment enhance the charge-screening effect after water penetration, maintaining a tighter gel matrix and confirming the results obtained for swelling in various concentrations of NaClO_4_. Additionally, the counterionic osmotic pressure difference between the gel matrix and the media decreases, making media penetration more difficult.^89^

### Antimicrobial Activity

Throughout our development of polymeric arsenical (nano)­materials, we have mitigated justifiable concerns regarding toxicity by highlighting that organic arsenicals are known to be less toxic than their inorganic counterparts^90^ and demonstrating that this extends to our polymeric arsenical platform through various *in vitro* cell viability experiments. For example, we have shown that nearly identical polymeric arsenical scaffolds exhibited limited toxicity via a standard XTT assay.^65^ Furthermore, hydrogels fabricated from these scaffolds support cell culture. In this work, to further dispel concerns around toxicity, we monitored the leaching of **P**_**As**_ from the hydrogel networks by UV–vis spectroscopy during swelling experiments. Based on a calibration curve, the amount of **P**_**As**_ released from **P**_**As**_ **-Pt@4** and **P**_**As**_ **-Pt@7** was <1 wt %, supporting the high stability of the networks as determined by rheology (Figure S13). Nevertheless, the biomedical use of these materials requires a comprehensive biocompatibility evaluation to ensure safety and functional integration *in vivo*. The antibacterial activity of the most stable hydrogels, **P**_**As**_ **-Pt@4** and **P**_**As**_ **-Pt@7** was tested against Gram-negative (*UPEC*, K12 MG1655) and Gram-positive (, ) (Figure S14A-D). Initially, a bacterial zone of inhibition assay was performed in the presence of **P**_**As**_ **-Pt@4** and **P**_**As**_ **-Pt@7** with K_2_PtCl_4_ employed as a Pt control (Figure 8). The **P**_**As**_ polymer scaffold and a blank NaClO_4_ solution were also tested as controls and were shown not to inhibit bacterial growth (Figure S15). For Gram-negative bacteria *UPEC* and *K12 MG1655*, the **P**_**As**_ **-Pt@7** hydrogels exhibited greater antibacterial activity compared to the **P**_**As**_ **-Pt@4** gels. The activity was comparable to that of the Pt^II^ control, suggesting that encapsulating Pt^II^ within the hydrogel matrix does not impair its antibacterial efficacy. This alludes to the potential of these and analogous materials as effective carriers for Pt^II^ drug delivery to inhibit the growth of Gram-negative bacteria. In contrast, for Gram-positive bacteria and , the **P**_**As**_ **-Pt@4** hydrogels exhibited greater antibacterial activity. Notably, in the case of , both hydrogels exhibited a significant increase in the zone of inhibition compared to the Pt control. This suggests that Pt^II^ alone may be less effective in inhibiting the growth of Gram-positive bacteria. However, the exact mechanism underlying the enhanced antibacterial activity under pH 4 conditions remains unclear and requires more detailed investigation.

**Figure 8:**
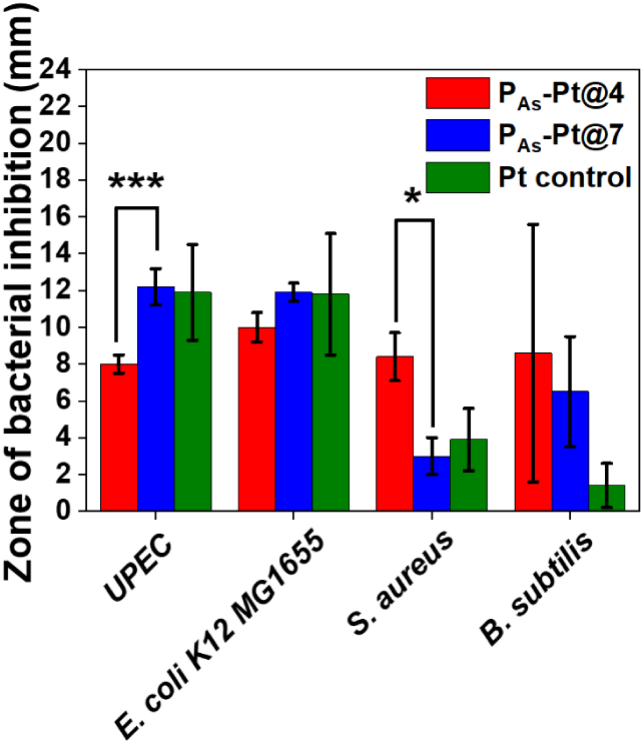
Antibiotic diffusion assay demonstrating the antibacterial activity of Pt control and **P**_**As**_ **@Pt4, P**_**As**_ **@Pt7** hydrogels against *UPEC*, *K12 MG1655* (Gram-negative) and , (Gram-positive) bacteria. Statistical significance is indicated as *p* = 0.05 (*), 0.01 (**), and 0.001 (***).

We further evaluated the bacterial viability of **P**_**As**_ **-Pt@2**, **P**_**As**_ **-Pt@4**, and **P**_**As**_ **-Pt@7** using the resazurin assay. This assay was chosen as it provides a direct and comparable assessment of bacterial viability within a short time frame (2 to 6 h)^91^ which also allowed for the assessment of the least stable **P**_**As**_ **-Pt@2** hydrogel. Notably, we observed a significant reduction in *K12MG1655* viability across all controls and hydrogel samples within 6 h, compared to the positive control (PC), as shown in Figure 9. displayed a similar depletion of bacterial viability. The antibacterial effects of the As and salt controls were contradictory to those observed in the antibiotic diffusion assay. For *UPEC* and , only the hydrogels demonstrated a significant decrease in bacterial viability compared to the PC, while the Pt control reduced bacterial viability only in the case of *UPEC*.

**Figure 9:**
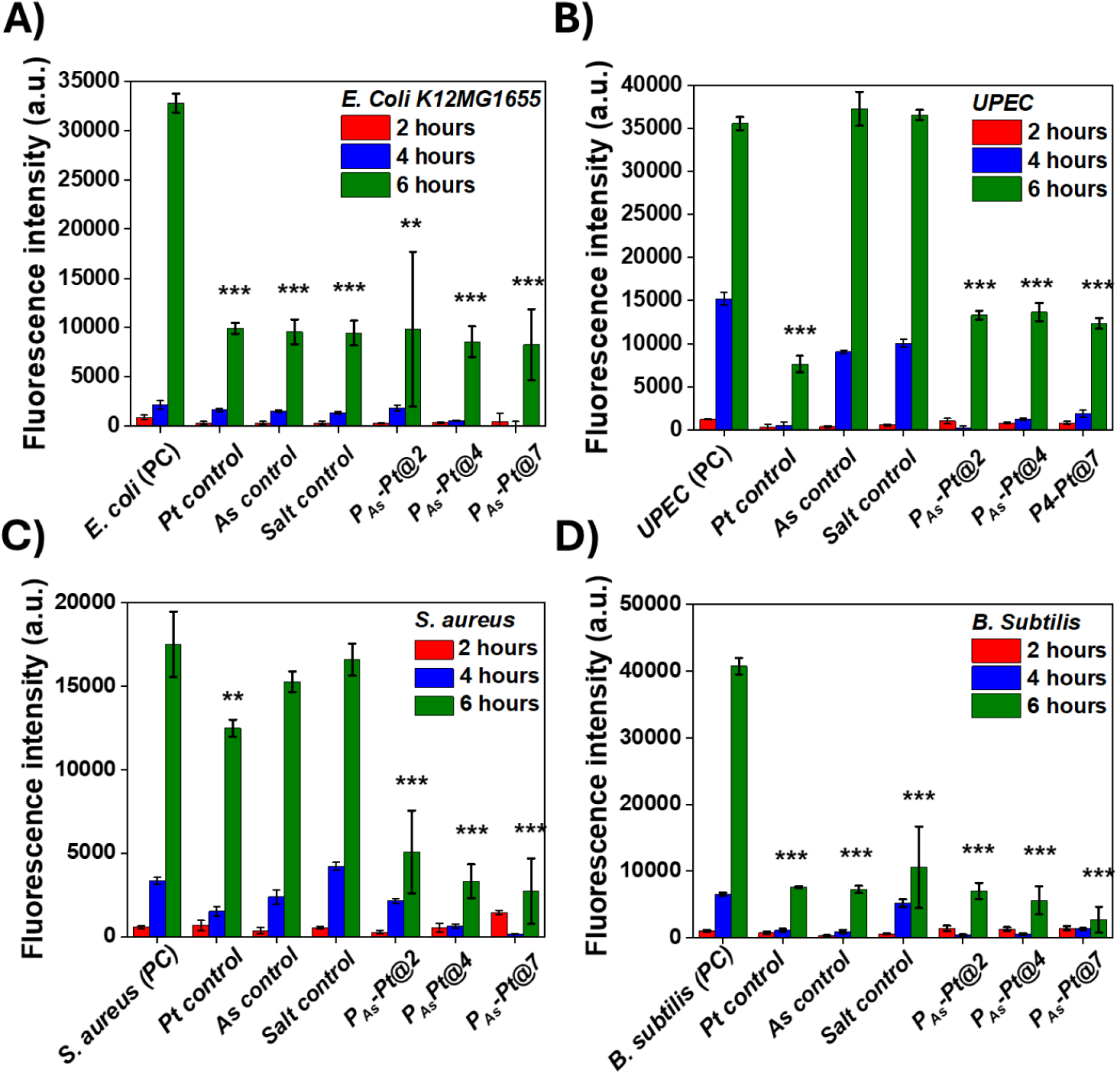
Bacterial viability assay for (A) *K12 MG1655* (B) *UPEC* (C) and (D) after 6 h of exposure to Pt control, Salt control, As control, and gels fabricated in different pH media. Statistical significance is indicated as *p* = 0.05 (*), *p* = 0.01 (**), and *p* = 0.001 (***), calculated between the 6 h PC and the other samples.

These findings suggest that hydrogels can exert antibacterial effects within a few hours of direct exposure to bacterial suspensions, with noticeable effects as early as 2 h and continuing up to 6 h. The differences in antibacterial effects observed between the antibiotic diffusion assay and the bacterial viability assay may be attributed to variations in bacterial growth patterns on solid substrates compared with those in solution.

Having shown that very little **P**_**As**_ is released from the hydrogel network (*vide supra*), a model Pt^II^-release assay was performed to gain a preliminary understanding of the origin of the antimicrobial activity observed. Hydrogels were prepared at the bottom of 15 mL Falcon tubes. After preparation, the hydrogels were centrifuged to ensure good adherence to the base of the tube, providing a consistent active surface for accurate comparison between samples. PBS (0.01 M, *I* = 0.15 M, pH 7.4) was carefully added to each tube, and the tubes were incubated in a shaking water bath at a constant temperature of 37 °C. Aliquots (200 μL) were periodically withdrawn for analysis by RP-HPLC to monitor the release of Pt^II^ (Figure S16). The hydrogels that retained structural integrity during swelling (**P**_**As**_ **-Pt@4, P**_**As**_ **-Pt@7**) exhibited a swelling-controlled release of Pt^II^ achieving 6.9% and 3.3% release of Pt^II^ over a week (Figure 10). In the case of **P**_**As**_ **-Pt@2**, a greater release of Pt^II^ was observed (14.0%). This enhanced release profile is likely due to the poor structural integrity of the **P**_**As**_ **-Pt@2** network (*vide supra*).

**Figure 10:**
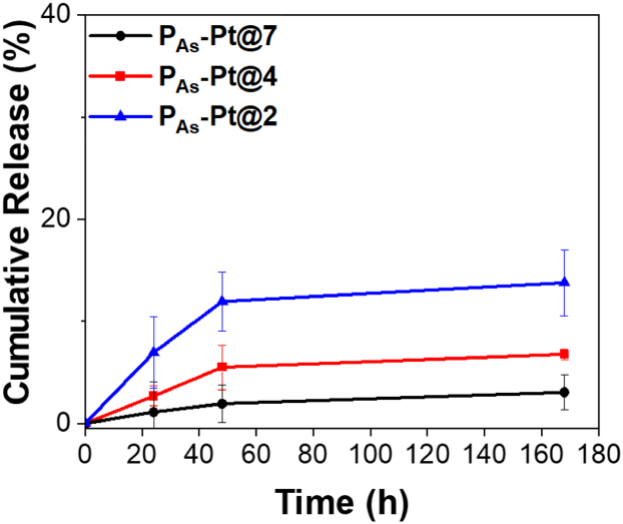
Release profile of Pt^II^ salts from **P**_**As**_ **-Pt@2**, **P**_**As**_ **-Pt@4**, and **P**_**As**_ **-Pt@7** gels in PBS (0.01 M, *I* = 0.15 M, pH 7.4) at 37 °C, monitored by RP-HPLC with UV detection at 320 nm.

While the exact nature of the coordination sphere of the Pt^II^ salts released is not known, our previous work confirms that the oxidation state of the Pt^II^ does not change during hydrogel formation.^37^ Furthermore, hydration of the Pt^II^ salts containing relatively labile ligands is known to occur over time scales relevant to these assays.^92^ Overall, considering the activity of the Pt^II^ control (K_2_PtCl_4_) in the antimicrobial assays and the detection of Pt^II^ release from the hydrogels, the origin of the antimicrobial activity of the arsenic–platinum hydrogels can be tentatively linked to the release of Pt^II^ salts from the networks. Further mechanistic investigation into the bioactivity of these materials is needed to fully understand the mechanisms behind the antibacterial activity of arsenic–platinum hydrogels and is subject to ongoing work in our program.

## Conclusions

Arsenic–platinum hydrogels derived from pDMAm_0.92_-co-AsAm_0.08_ (**P**_**As**_) with K_2_PtCl_4_ were fabricated at different pH values. UV–vis spectroscopy revealed efficient gelation only at pH 4 and 7 at 2.5 wt %, reflecting the pH-sensitive nature of these materials. ^1^H NMR, ^195^Pt NMR, and FT-IR analysis showed that, in its acid form, arsenic acid forms weaker As–Pt interactions, impacting hydrogel integrity and dynamic behavior. Oscillatory rheology revealed that cross-linking density is enhanced when pH > p*K*_a1_ of the arsenic acid pendant group, with *G*’ displaying a pH-dependent maximum before decreasing in alkaline environments due to platinum hydroxide formation. All hydrogels exhibited macroscopic self-healing, as evidenced by rheological recovery after mechanical damage. The looser and more flexible **P**_**As**_ **-Pt@2** network exhibited excellent resilience upon stretching compared to the more rigid **P**_**As**_ **-Pt@4** and **P**_**As**_ **-Pt@7**. **P**_**As**_ **-Pt@4** and **P**_**As**_ **-Pt@7** displayed greater stability and swelling, while swelling was reduced in PBS, consistent with charge screening effects. Antimicrobial testing against Gram-negative (*UPEC*, *K12 MG1655*) and Gram-positive (, ) bacteria demonstrated comparable or improved activity relative to Pt controls, with no strong pH dependence observed. Further studies of the underlying antibacterial mechanisms are currently ongoing. Overall, this research highlights the robust and adaptable nature of these hydrogels, where simple environmental factors such as pH and ionic strength can be used to tune their mechanical properties. The translation of the system into pH-responsive nanoparticle formulations combining arsenic and platinum could enable the development of new therapies, leveraging Pt–As synergy against both infectious and noncommunicable diseases.

## Supplementary Material







## Notes

Raw data sets are available from the authors upon request. Additional experimental analysis, including supportive figures, tables, and images; NMR; SEC; SEM/EDX; amplitude frequency sweeps; swelling ratios; macroscopic self healing; disk diffusion assays; HPLC chromatograms; calibration curves; and UV–vis (PDF)
Video recordings, demonstrating the mechanical resilience of the self-healed **P**_**As**_ **-Pt@4** hydrogel upon stretching (SV1) (MP4)
Video recordings, demonstrating the mechanical resilience of the self-healed **P**_**As**_ **-Pt@7** hydrogel upon stretching (SV2) (MP4)

## §

Medherant Ltd., The Venture Center, University of Warwick Science Park, Coventry, CV4 7EZ
