# Supplementary material for: The Effect of pH during Fabrication of Platinum-Containing Polymeric Arsenical Hydrogels
Source: Macromolecules. 2025 Jul 17;58(15):7709–21. doi: 10.1021/acs.macromol.5c01254 (PMC12356066; doi:10.1021/acs.macromol.5c01254)
Supplement: Supplementary file 1 [file ma5c01254_si_001.pdf]

pH-Responsive Antimicrobial Hydrogels Incorporating Platinum-Containing Polymeric Arsenicals

Alexandros Magiakos,[a] Spyridon Efstathiou,[a] Evelina Liarou,[a] Andrea Dsouza,[b] Chrystala Constantinidou[b] and Paul Wilson[a]*

[a]Department of Chemistry, University of Warwick, Coventry, CV4 7AL, UK

[b]Warwick Medical School, University of Warwick, Coventry CV4 7AL, UK

*Corresponding Author, Email: p.wilson.1@warwick.ac.uk

SUPPORTING INFORMATION

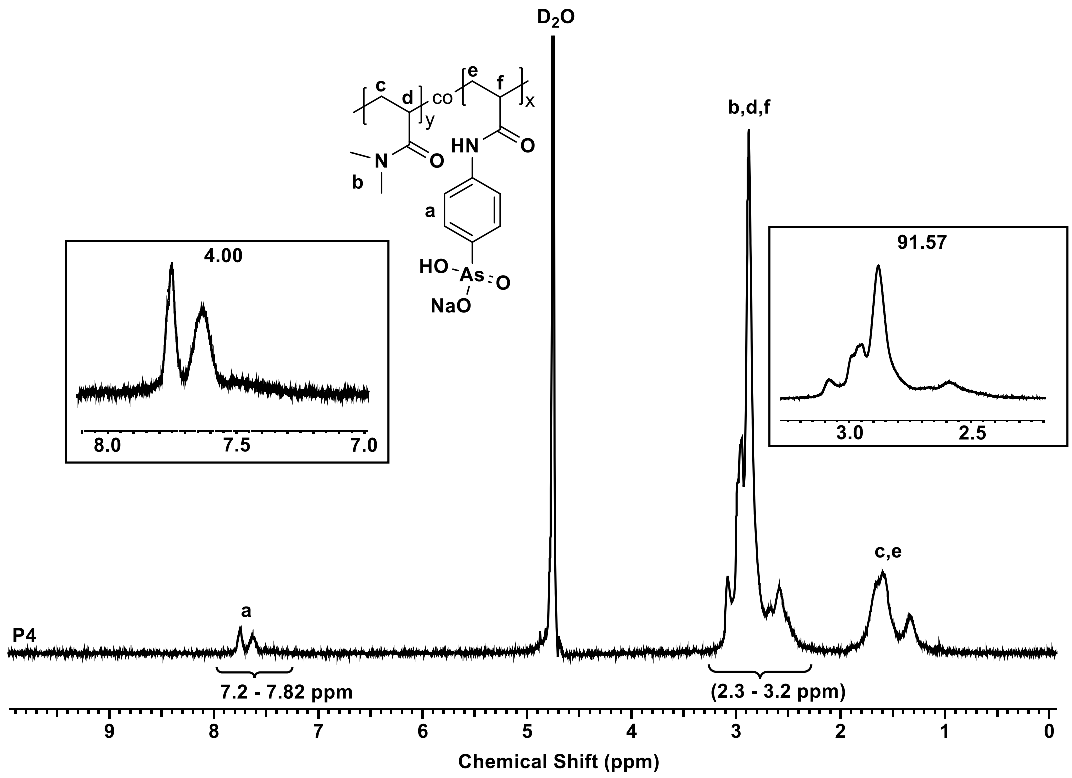

Figure S1A. ^{1}H NMR spectra (400 MHz, D_2O) of $\mathbf{P_{As}}$ polymeric arsenical scaffold. Calculation of AsAm content with respect to DMA followed the equation:

$$\frac{\int[[M]_{AsAm}+7[M]_{DMAm}]_{2.3-3.2\,ppm}-\frac{\int 4[[M]_{AsAm}]_{7.2-7.82\,ppm}}{4}}{7}=[M]_{DMAm}$$

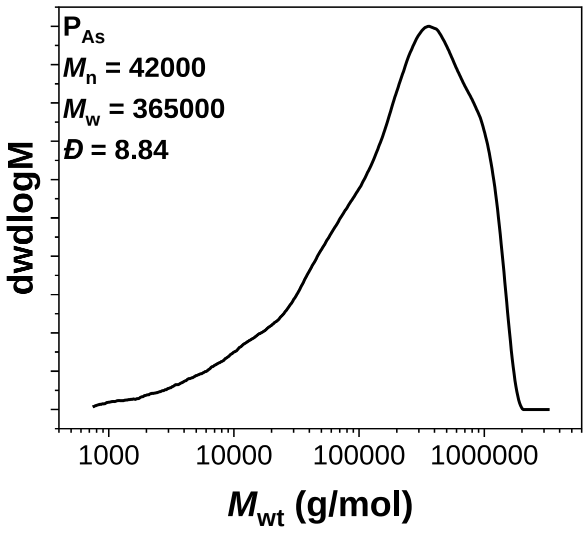

Figure S1B. Aqueous SEC of $\mathbf{P_{As}}$ polymeric arsenical.

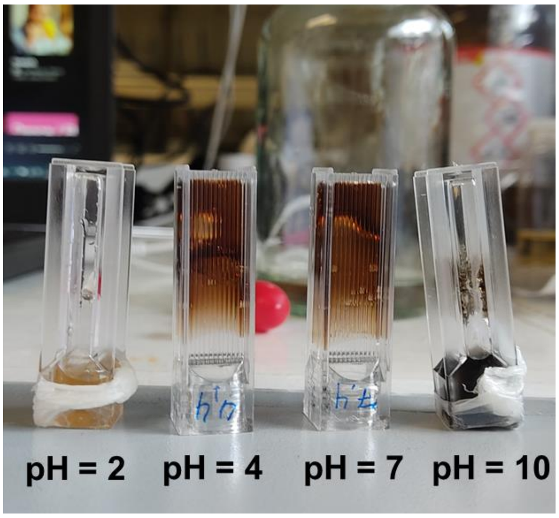

Figure S2. Image of $\mathbf{P_{As}}$/K_2PtCl_4 solutions (2.5 wt % at different pH conditions after 24 h at 50 °C. The mixtures at pH 2 and pH 10 fail to form a gel when concentration 2.5 wt %.

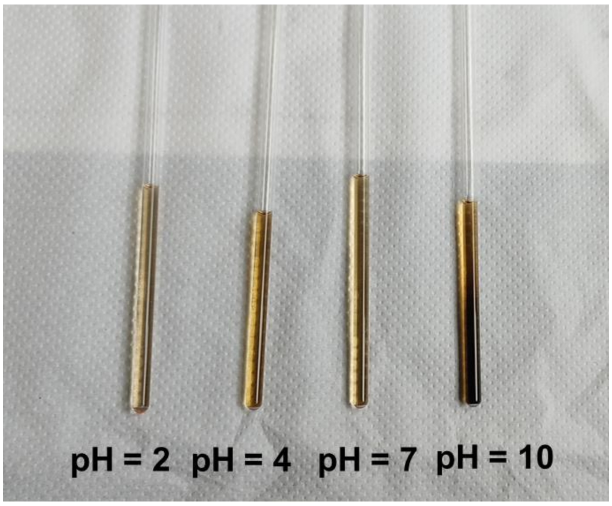

Figure S3. Hydrogels formed upon mixing 10 wt % $\mathbf{P_{As}}$ polymer solutions with K_2PtCl_4 ([As]/[Pt] = 1) at different pH conditions inside the NMR tubes (*in situ)* and stored in the dark at ambient temperature for 5 days prior to analysis by NMR.

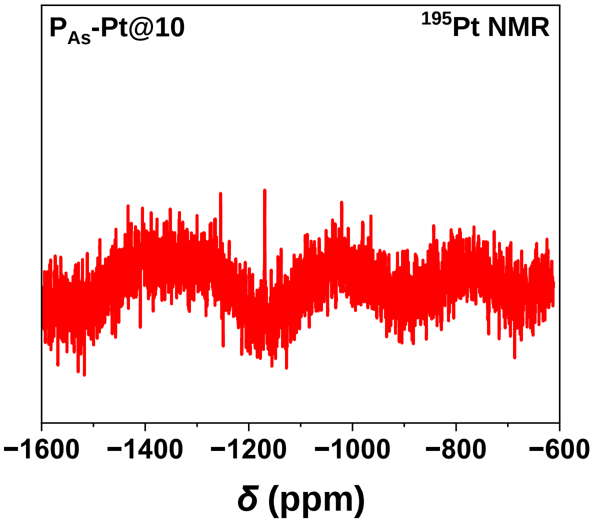

Figure S4. ^{195}Pt NMR spectrum (600 MHz, D_2O) of the **P_{As}-Pt@10** in the range of -1600 to -600 ppm.

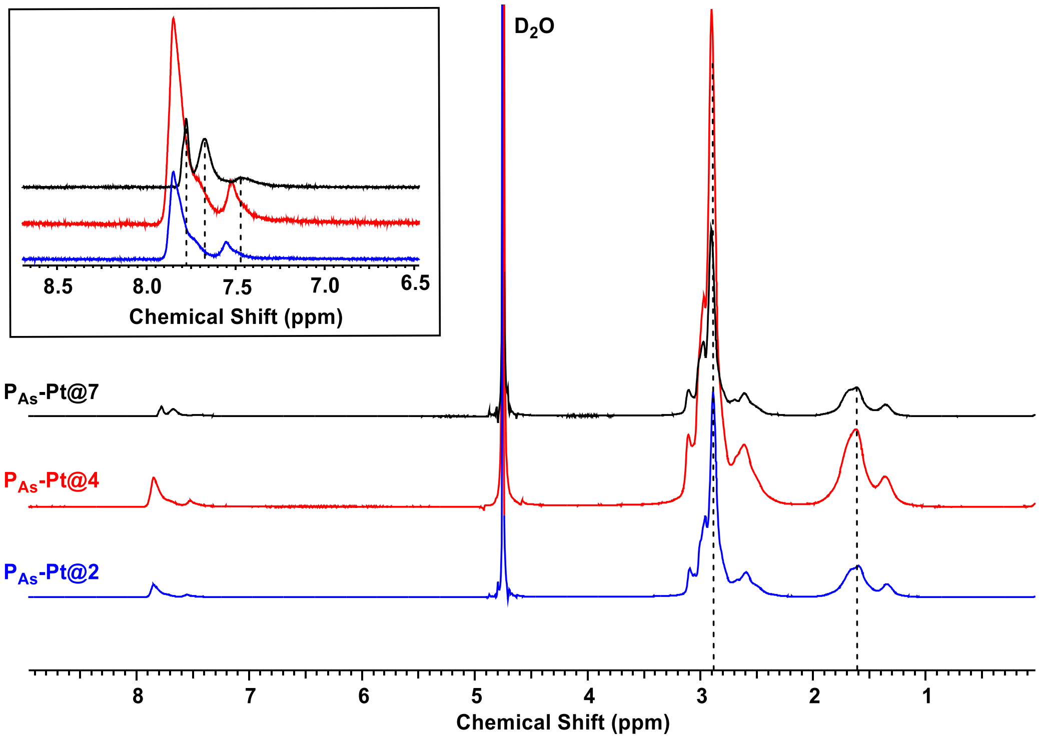

Figure S5. ^{1}H NMR (400 MHz, D_2O) of the corresponding hydrogels showing the downfield shift in AsAm side chain peaks upon pH decrease.

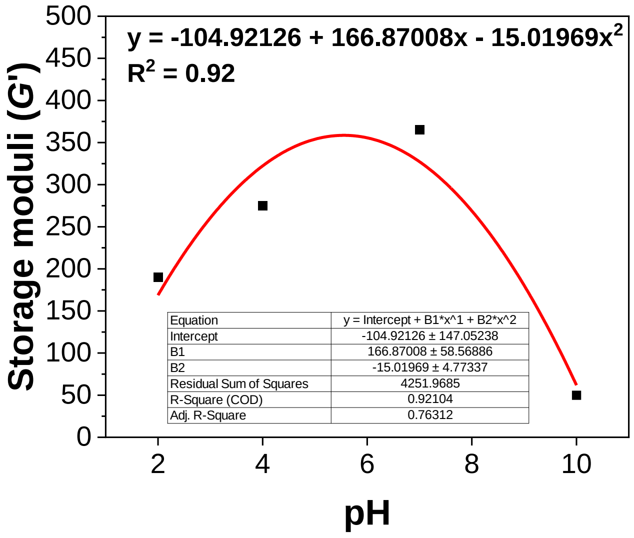

Figure S6. *G'* at angular frequency of ω = 1 rad.s^{-1} as a function of pH with Red Gaussian's fitting formula and standard errors for each parameters.

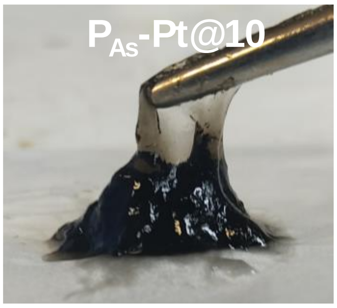

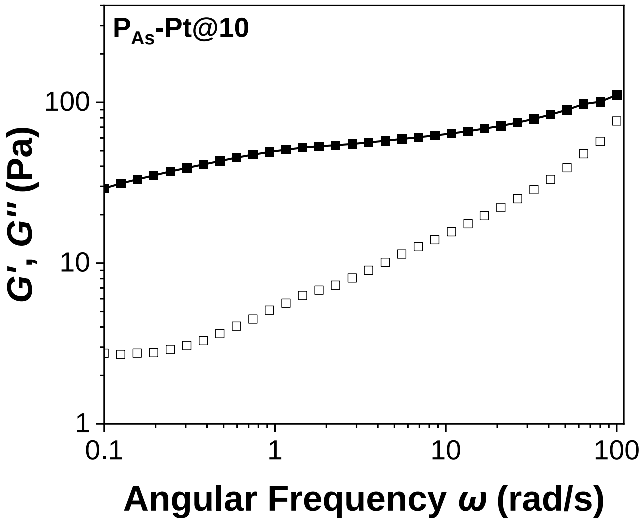

Figure S7. P_{As}-Pt@10 hydrogel (up) and its frequency sweep curve (ω = 0.1-100 rad.s^{-1}) at 25 ^{o}C and at a constant strain of γ = 10.0% obtained from plate-to-plate oscillatory rheology (down). Storage moduli (*G'* , filled symbols) and loss moduli (*G''*, unfilled symbols).

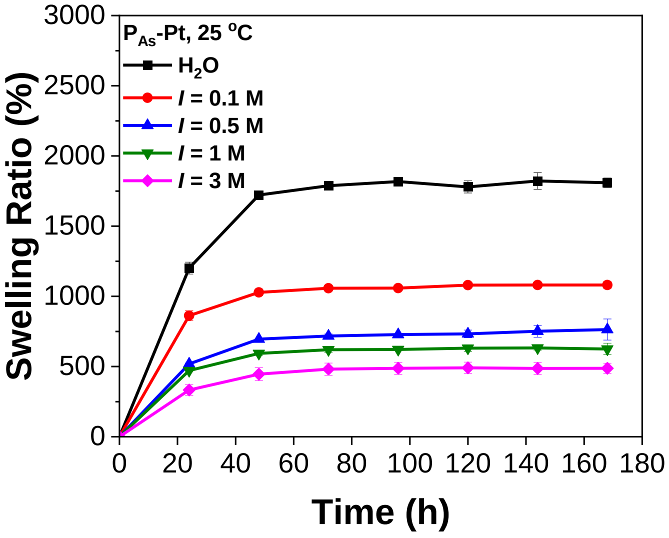

Figure S8A. Swelling ratio curves of **P_{As}-Pt** fabricated in milli-Q water immersed in aqueous solutions of different ionic strengths.

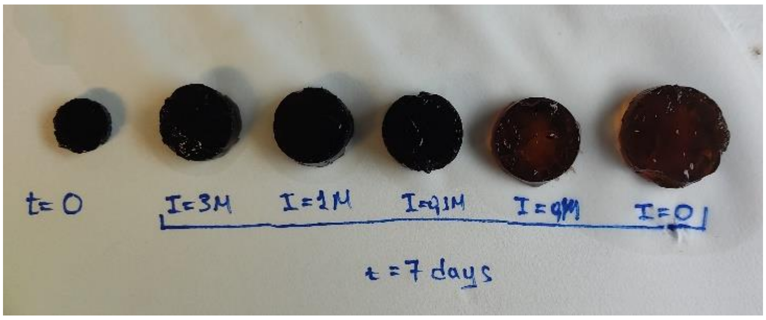

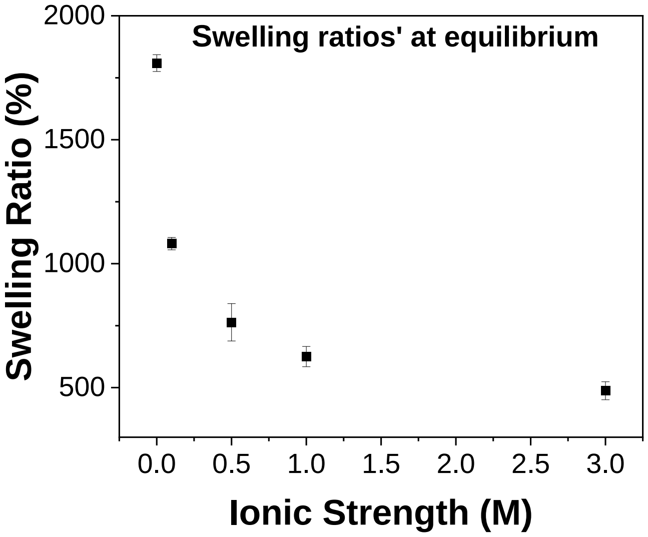

Figure S8B. Comparison of the sizes of **P_{As}-Pt** gels at swelling equilibrium (168 h) as a function of ionic strength (up). Swelling ratio values at equilibrium (168 h) (down).

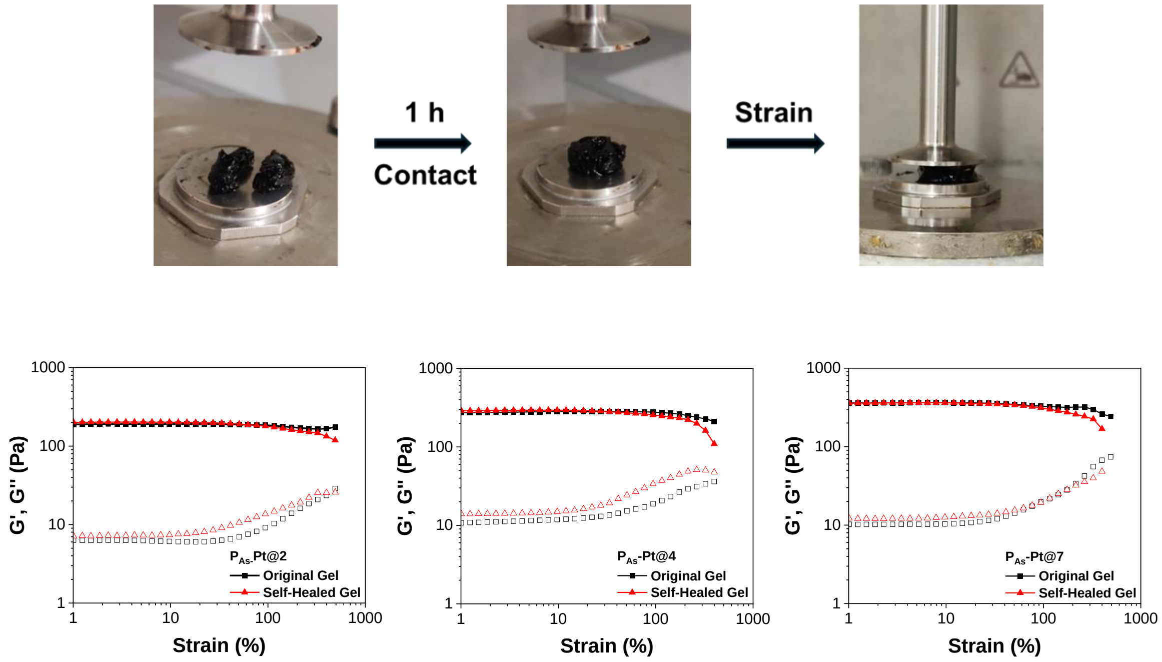

Figure S9. Representative demonstration of self-healing potential of **P_{As}-Pt@2, P_{As}-Pt@4** and **P_{As}-Pt@7** hydrogels. Freshly prepared gels were cut in half and allowed to recombine in a humidity chamber for 1 h. Recycled amplitude sweeps were performed before and after the healing process, with both *G'* and *G''* values showing comparable results to the original gels.

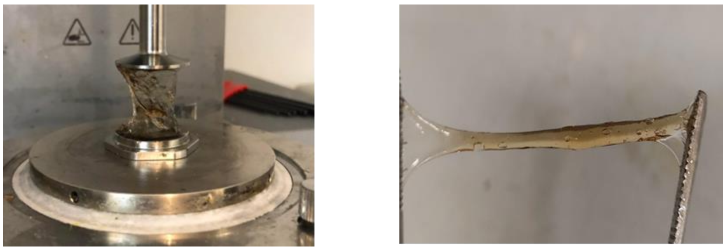

Figure S10. Demonstration of the resilience to stretching for **P_{As}-Pt@2** self-healed hydrogel.

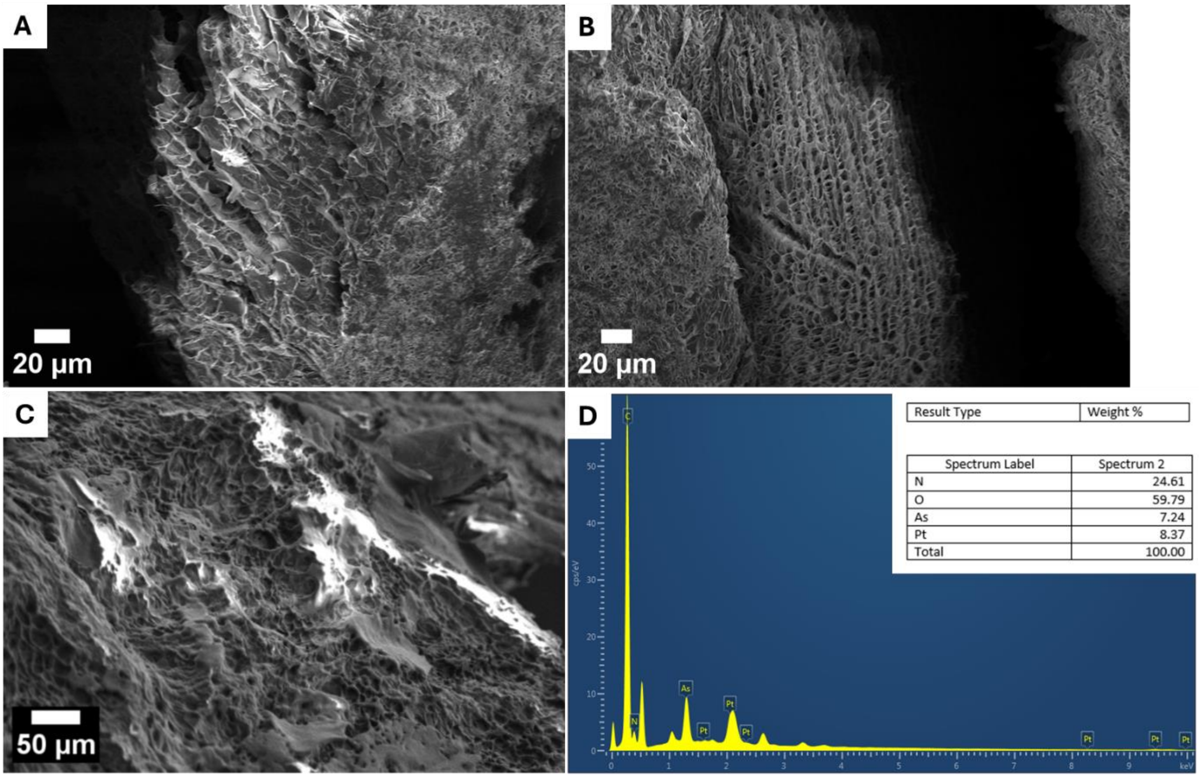

Figure S11A. (A-C) Scanning electron microscopy (SEM) image of **P$_{As}$-Pt@2** dried gel, showing the surface and pore structure. (D) SEM/EDX spectrum for the image C. Scale bars represent 50 μm and 20 μm.

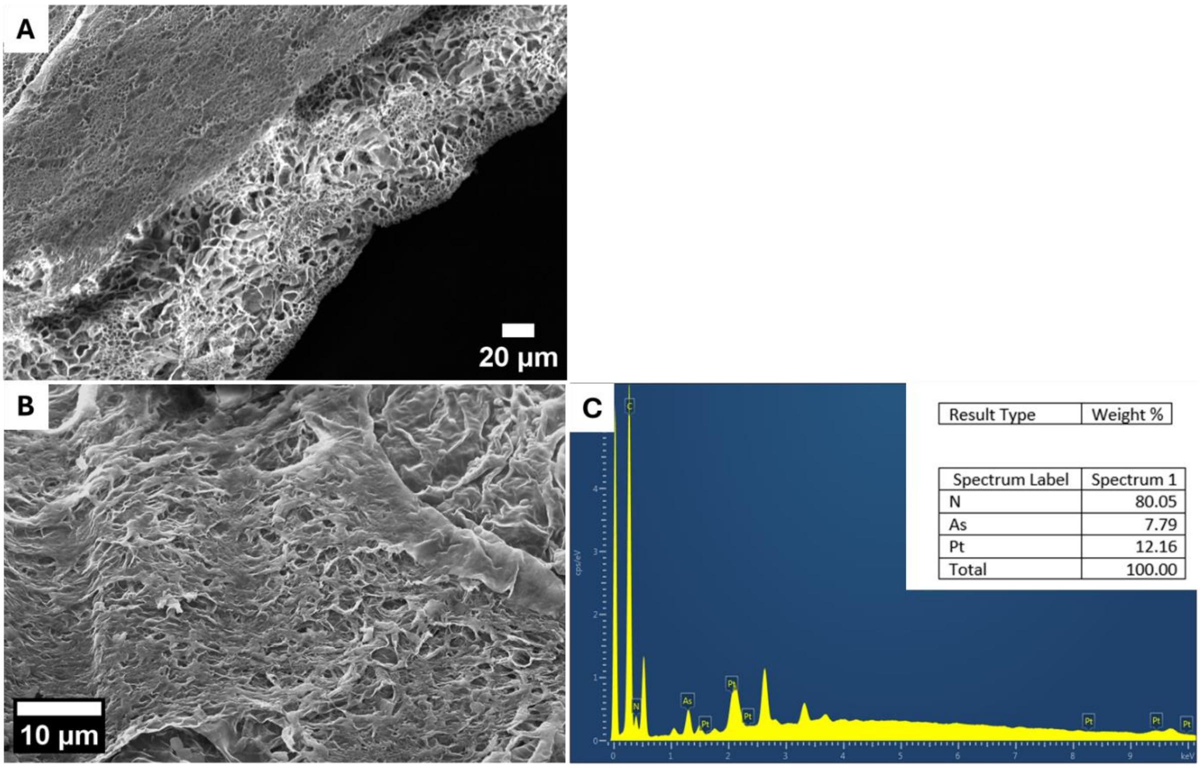

Figure S11B. (A-B) Scanning electron microscopy (SEM) image of **P$_{As}$-Pt@4** dried gel, showing the surface and pore structure. (C) SEM/EDX spectrum for the image B. Scale bars represent 20 µm and 10 µm.

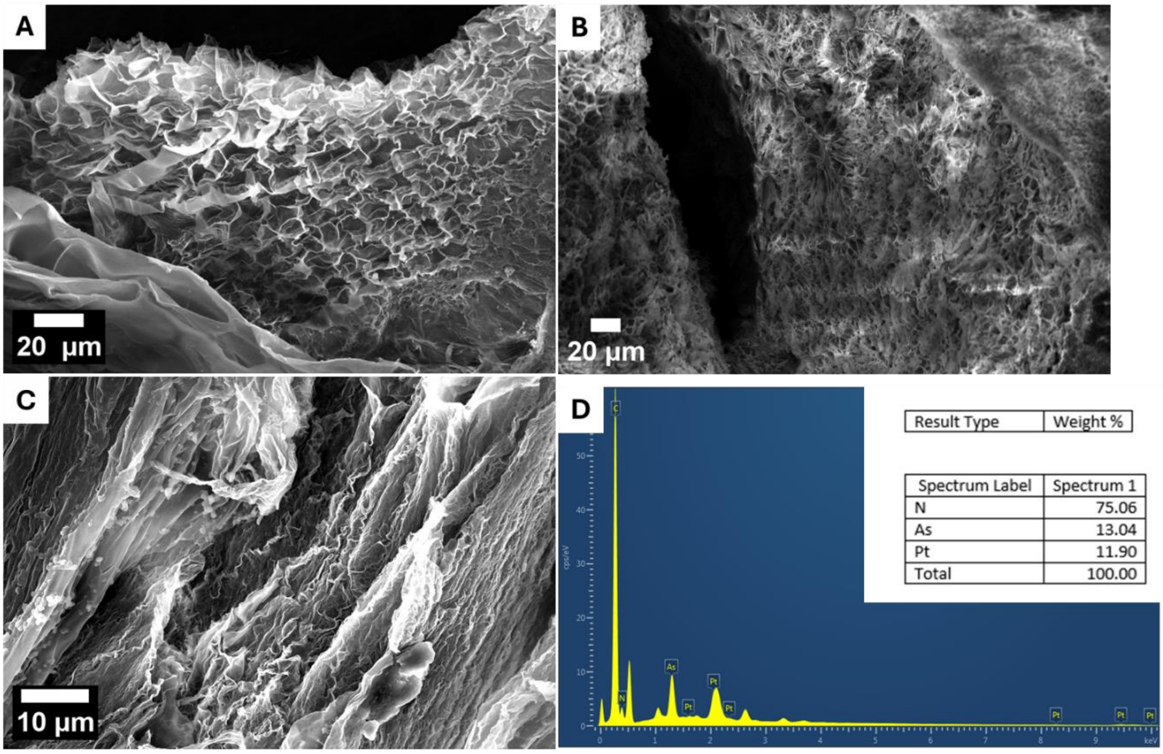

Figure S11C. (A-C) Scanning electron microscopy (SEM) image of **P$_{As}$-Pt@7** dried gel, showing the surface and pore structure. (D) SEM/EDX spectrum for the image C. Scale bars represent 20 μm and 10 μm.

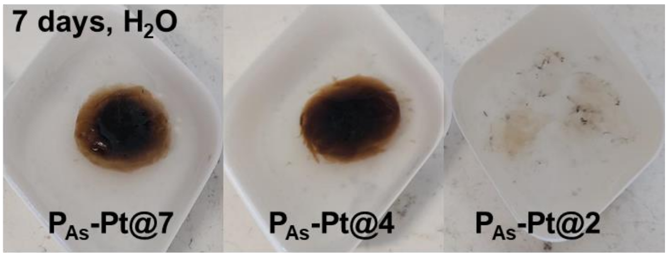

Figure S12. Images of **P$_{As}$-Pt@7**, **P$_{As}$-Pt@4** and **P$_{As}$-Pt@2** hydrogels at 7 days of swelling in milli-Q water.

Table S1. Swelling behavior of **P_{As}-Pt@4 and P_{As}-Pt@7** hydrogels in milli-Q water and PBS as a function of time, expressed as the degree of swelling $(W_s - W_o)/W_o \times 100$, rounded to the nearest 5 or 10.

Media	Hydrogel	24 h	48 h	72 h	96 h	120 h	144 h	168 h
H_2O	**P_{As}-Pt@4**	500	900	1285	1645	1780	1820	1825
H_2O	**P_{As}-Pt@7**	455	860	1165	1465	1570	1590	1595
PBS	**P_{As}-Pt@4**	380	690	950	1050	1145	1170	1190
PBS	**P_{As}-Pt@7**	350	615	820	885	885	895	905

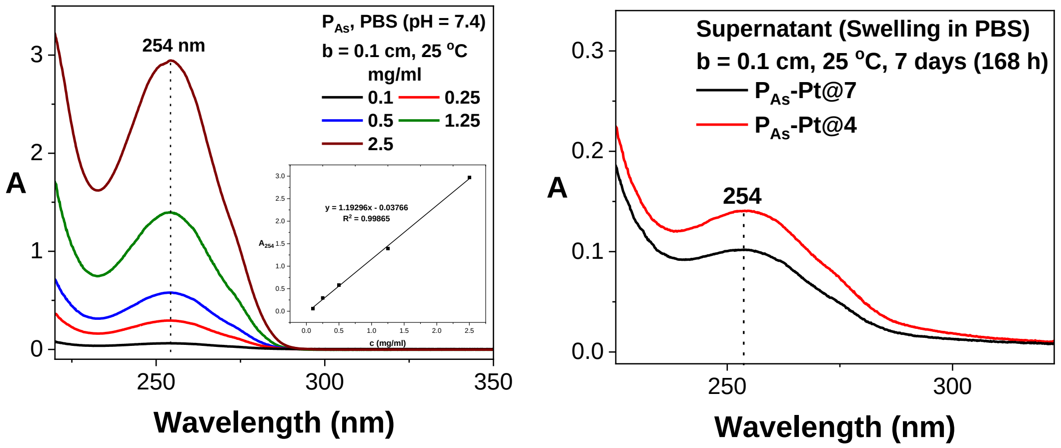

Figure S13. Calibration curve of UV–Vis absorption for **P_{As}** polymer in PBS (pH 7.4), constructed using standard solutions (mg/mL) (left). UV-Vis absorption of the supernatant collected during swelling studies of **P_{As}-Pt@4** and **P_{As}-Pt@7** hydrogels in PBS after 7 days (168 h) (right). **P_{As}** leaching was quantified using the calibration curve and expressed as weight percent (wt %).

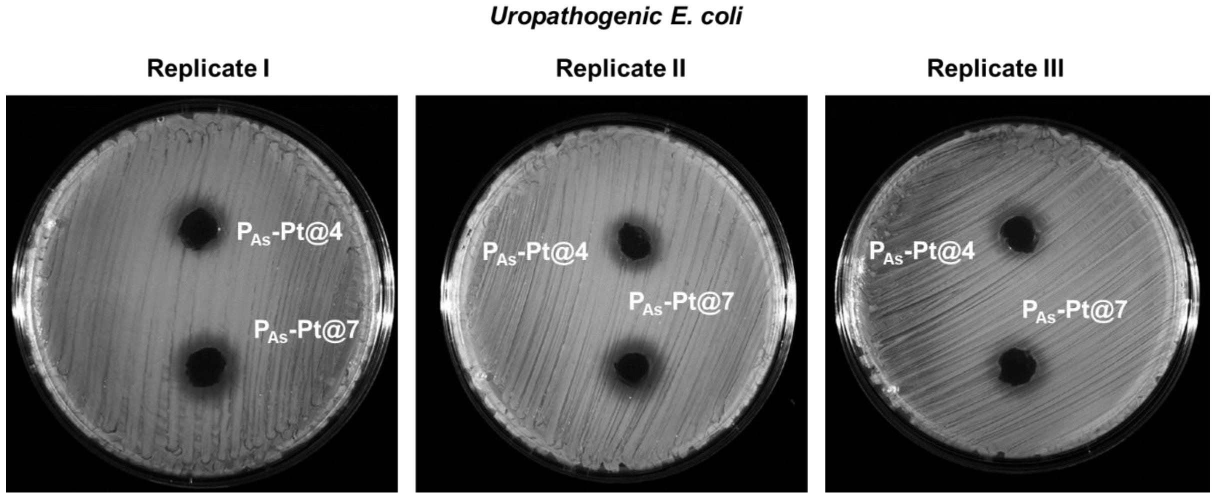

Figure S14A. Antibiotic diffusion assay for **P_{As}-Pt@7** and **P_{As}-Pt@4** gels against *UPEC.*

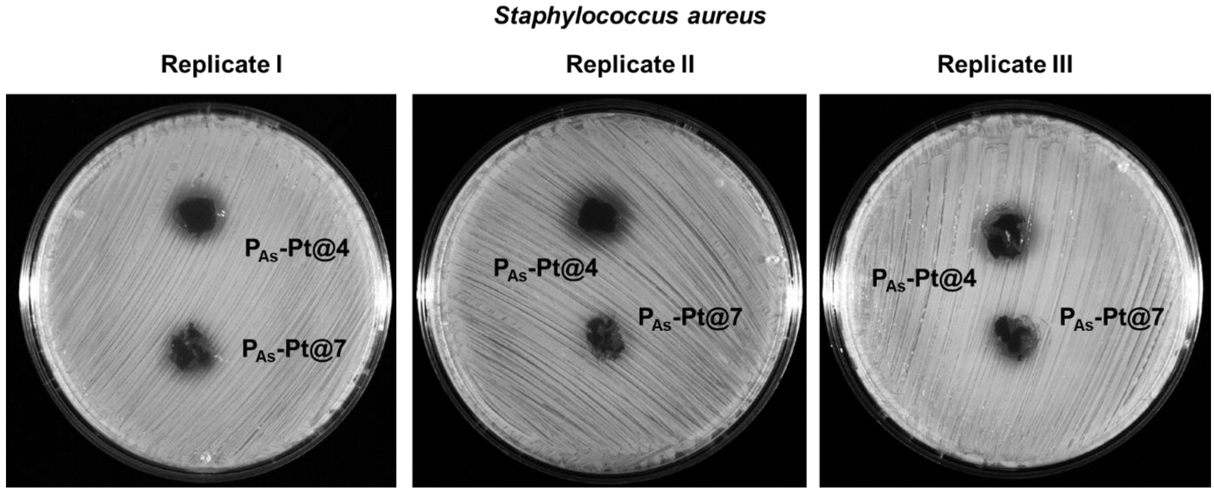

Figure S14B. Antibiotic diffusion assay for **P_{As}-Pt@7** and **P_{As}-Pt@4** gels against *S. aureus.*

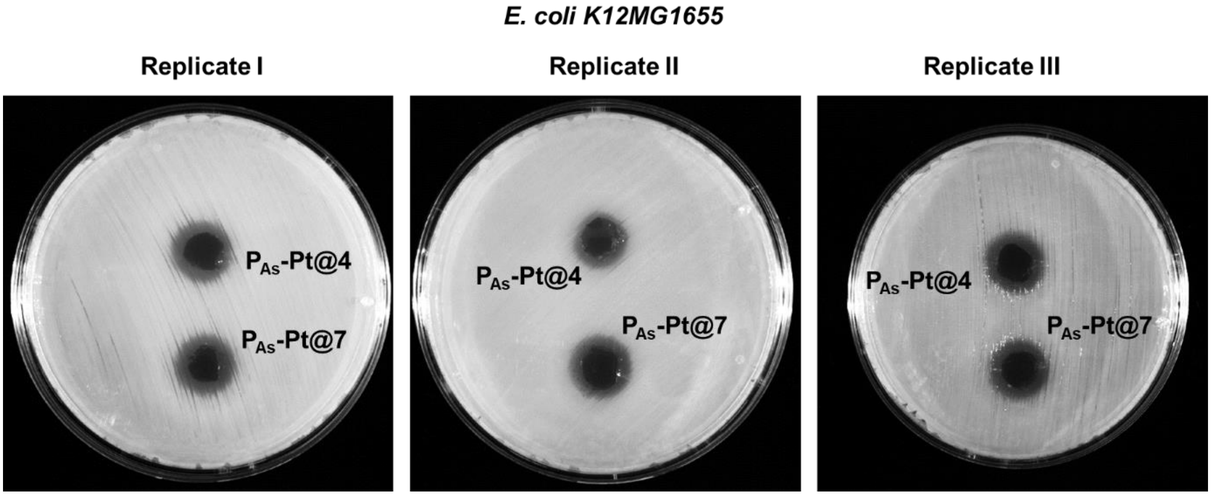

Figure S14C. Antibiotic diffusion assay for **P_{As}-Pt@7** and **P_{As}-Pt@4** gels against *E. coli K12MG1655.*

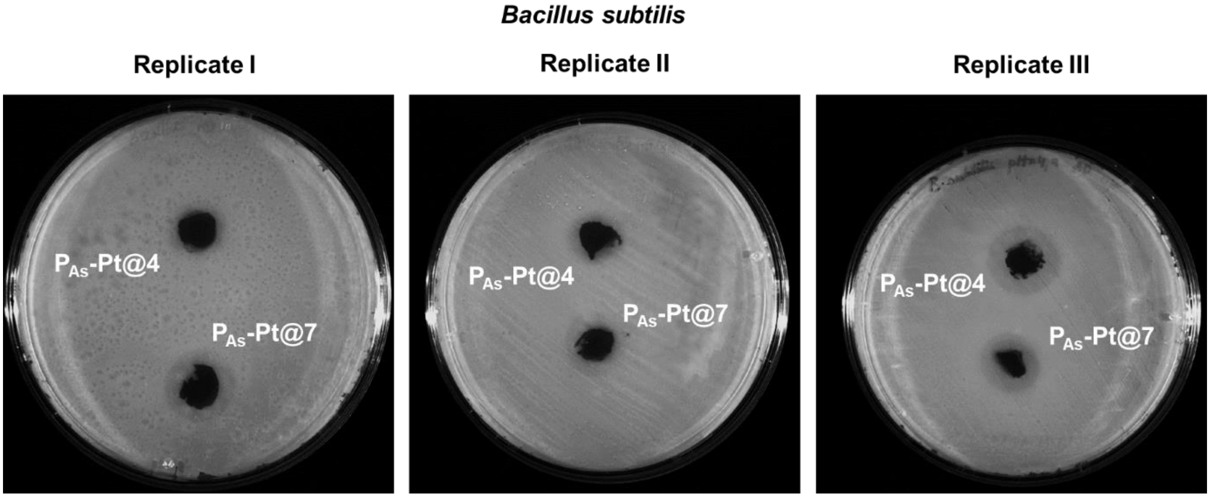

Figure S14D. Antibiotic diffusion assay for **P_{As}-Pt@7** and **P_{As}-Pt@4** gels against *B. subtilis.*

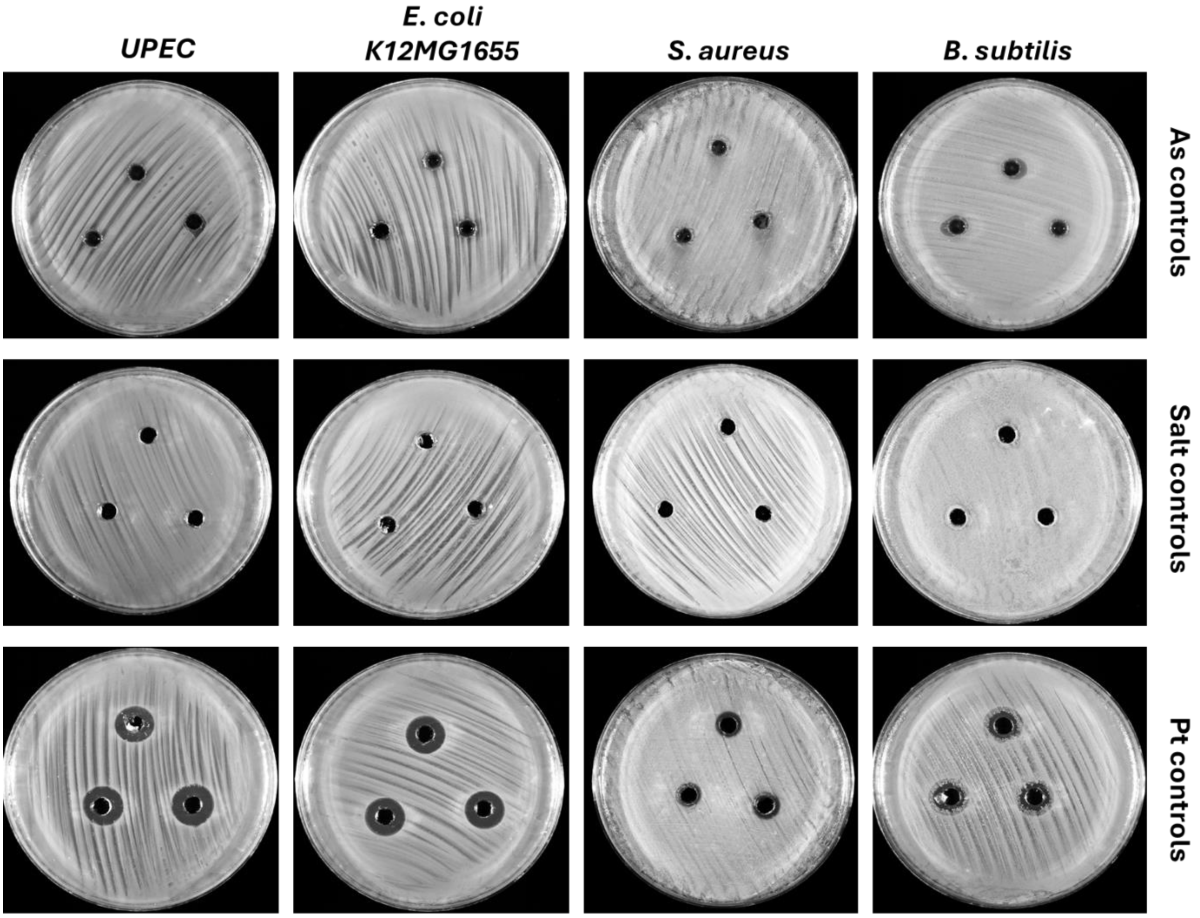

Figure S15. Antibiotic diffusion assay for As, salt, and Pt controls against *UPEC*, *E. Coli K12MG1655*, *S. aureus* and *B. subtilis*. *As controls*: **P4** polymer scaffold aqueous solution at a concentration of 100 mg.mL^{-1}. *Salt controls*: Sodium perchlorate ($NaClO_4$ 0.1 M) salt solution. *Pt controls*: Platinum (Pt^{II}) aqueous solution of 30 mM K_2PtCl_4, matching the Pt^{II} concentration in the 10 wt % gel matrix.

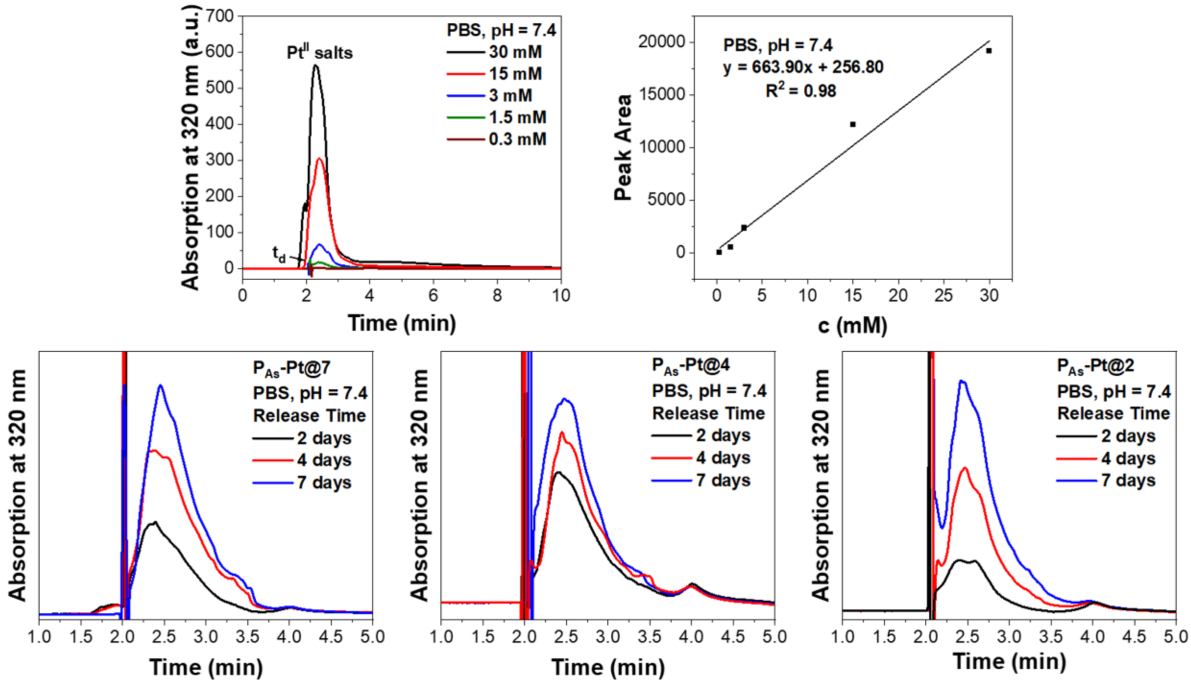

Figure S16. HPLC calibration curves for Pt^{II} salts in PBS (0.01 M, I = 0.15M, pH =7.4), constructed using standard K_2PtCl_4 solutions (0.3 - 30 mM) incubated for 5 days (25 ºC) and UV detection at 320 nm (top). Release profiles from **P_{As}-Pt@2**, **P_{As}-Pt@4** and **P_{As}-Pt@7** hydrogels over 7 days (168 h) in PBS at 37 °C, determined by the signal peaks in reverse-phase HPLC (bottom).
